# Metallocene-Filled Single-Walled Carbon Nanotube Hybrids

**DOI:** 10.3390/nano13040774

**Published:** 2023-02-19

**Authors:** Marianna V. Kharlamova, Christian Kramberger

**Affiliations:** 1 Centre for Advanced Materials Application (CEMEA), Slovak Academy of Sciences, Dúbravská cesta 5807/9, 845 11 Bratislava, Slovakia; 2 Faculty of Physics, University of Vienna, Boltzmanngasse 5, 1090 Vienna, Austria

**Keywords:** metallocene, carbon nanotube, electronic properties, Raman spectroscopy, near edge X-ray absorption fine structure spectroscopy, photoemission spectroscopy, optical absorption spectroscopy

## Abstract

In this paper, the growth mechanism, structure, growth processes, growth kinetics, and optical, vibronic and electronic properties of metallocene-filled single-walled carbon nanotubes (SWCNTs) are considered. A description of the procedures used to fill the nanotubes is provided. An investigation of doping effects on metallicity-mixed SWCNTs filled with metallocenes by Raman spectroscopy, near edge X-ray absorption fine structure spectroscopy, photoemission spectroscopy, and optical absorption spectroscopy is described. The studies of doping effects on metallicity-sorted SWCNTs filled with metallocenes are discussed. Doping effects in metallicity-mixed and sorted SWCNTs upon the chemical transformation of encapsulated molecules are analyzed. A discussion of the modification of the electronic properties of filled SWCNTs is presented. Applications of metallocene-filled SWCNTs in electrochemistry, thermoelectric power generation, chemical sensors, and magnetic recording are discussed.

## 1. Introduction

Metallocene-filled SWCNTs are very promising for application [1,2,3,4,5,6,7,8,9,10]. The first molecule introduced inside SWCNTs was C_60_ fullerene [11]. Later on, many scientific groups synthesized similar nanostructures, as well as filling SWCNTs with other fullerenes C_n_ (n = 70, 78, 80, 82, 90) [12,13,14,15,16,17,18,19,20,21,22,23,24,25,26,27,28,29,30,31,32,33], endohedral fullerenes (M_n_@C_m_, where M = Ca, Ti, Sc, La, Ce, Sm, Gd, Dy, n = 1, 2, m = 80, 82, 84, 92 [15,20,24,34,35,36,37,38,39,40,41,42,43,44,45,46]), exohedral fullerenes (C_60_O [47], C_60_Cs [48], complexes of C_60_ with Re and Os carbonyls [49,50,51]), fullerenes modified with functional groups (C_61_(COOH)_2_, C_61_(COOC_2_H_5_)_2_ [19,52]), metallocenes (ferrocene (FeCp_2_) [53,54,55,56,57,58,59,60,61,62,63,64,65,66,67,68,69], cobaltocene (CoCp_2_) [3,70,71,72], nickelocene (NiCp_2_) [73,74,75,76], cerocene (CeCp_3_) [77,78]), organic molecules (platinum (II) [64,79,80], nickel (II) [81] and cobalt (II) [82] acetylacetonates (Pt(acac)_2_, Ni(acac)_2_ and Co(acac)_2_, respectively), and others [18,83,84,85,86,87]).

SWCNTs were first filled with metallocenes in 2005: in Refs. [54,70], ferrocene (FeCp_2_) and cobaltocene (CoCp_2_) were incorporated, accordingly In 2009, cerocene (CeCp_3_) molecules were filled inside SWCNTs [77]. The formation of double-walled carbon nanotubes (DWCNTs) from metallocene-filled SWCNTs was first reported for ferrocene in 2008 [60]. In 2015, nickelocene molecules were encapsulated inside SWCNTs and DWCNTs were formed [73]. It was shown that the filled SWCNTs have interesting electronic properties and that thermal treatment leads to the growth of carbon nanotubes with a modified precisely tailored electronic structure that is important for their application.

**Figure 1:**
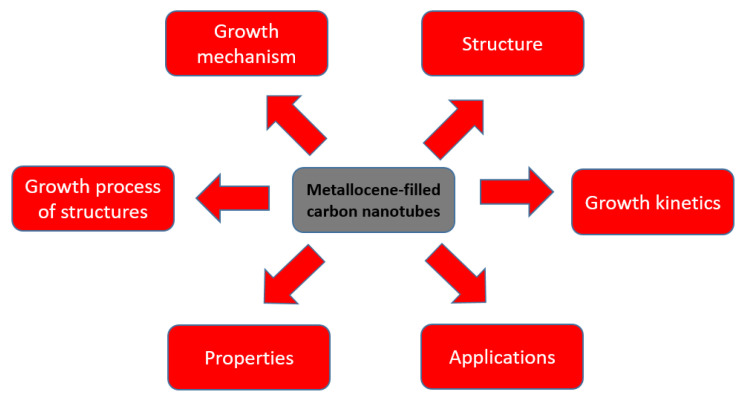
The schematics of investigations, and applications of metallocene-filled carbon nanotubes.

Metallocene-filled carbon nanotubes attract the attention of researchers for six reasons (Figure 1). First is the growth mechanism of carbon nanotubes with metal carbides as catalysts of the synthesis process. The nanoparticles can be in liquid or solid state during the synthesis process [88,89,90,91,92,93,94,95]. The chemical state of the nanoparticles can be carbidic or metallic [96,97,98,99,100,101,102,103,104,105,106,107]. The carbon nanotubes can grow in base- or tip-growth modes [108,109,110,111,112]. There is also the distinction between tangential and perpendicular growth modes [113,114,115].

Secondly, the structure of SWCNTs filled with metallocenes attracts interest because it is important to fill the bundles of nanotubes. Scanning electron microscopy (SEM) [116] and transmission electron microscopy (TEM) [72,74,75,116,117] are used. The chemical state of the filler is confirmed by energy dispersive analysis (EDX) [116]. The structure of DWCNTs formed as a result of the annealing of metallocene-filled SWCNTs is characterized [116].

Thirdly, the growth process of carbon nanotubes and other nanostructures is studied with microscopy. It is shown that pyrolysis of metallocenes leads to the formation of metal-filled carbon nanotubes [118]. The treatment of ferrocene at different pressures and temperatures was demonstrated to result in the synthesis of amorphous carbon, microparticles, nanotubes, microcones, and spirals [119].

Fourthly, the growth kinetics of carbon nanotubes inside metallocene-filled SWCNTs attracts huge interest [72,75,120]. The growth process of carbon nanotubes is characterized in two stages, with carbidic and metallic catalytic particles, accordingly Each stage is characterized with activation energy and growth rate [75]. They are dependent on the diameter and chiral angle of the nanotubes, and the metal type [120].

Fifthly, the characterization of the optical, vibronic, and electronic properties of SWCNTs filled with metallocenes is considered. Raman spectroscopy, near edge X-ray absorption fine structure spectroscopy (NEXAFS), photoemission spectroscopy (PES), and optical absorption spectroscopy (OAS) are performed [121,122,123,124,125,126,127,128,129,130,131,132,133,134,135,136,137,138,139,140,141,142,143,144,145,146,147,148,149,150,151,152,153,154,155,156,157,158,159,160,161,162,163,164,165,166,167,168,169,170,171]. The investigation of doping effects in metallicity-sorted SWCNTs filled with metallocenes is discussed [67].

Sixthly, applications of metallocene-filled SWCNTs attract interest. Among them, applications in electrochemistry [172], thermoelectric power generation [3], chemical sensors [173], and magnetic recording [174,175,176,177] are considered to be promising. For these applications, carbon nanotubes with homogeneous properties are needed. The listed applications are most viable. Other possible applications are in solar cells and light emission devices.

The aim of this paper is to review the growth mechanism, structures, growth processes, growth kinetics, and optical, vibronic, and electronic properties of metallocene-filled SWCNTs.

In Section 2, the growth mechanism of carbon nanotubes is discussed. In Section 3, the structure of SWCNTs filled with molecules is characterized. In Section 4, the growth process of carbon nanotubes and other structures is discussed. In Section 5, the growth kinetics of carbon nanotubes are considered. In Section 6, the optical, vibronic, and electronic properties of SWCNTs filled with molecules are characterized. In Section 7, the investigation of doping effects in metallicity-sorted SWCNTs filled with molecules is discussed. In Section 8, investigations of doping effects in metallicity-mixed and -sorted SWCNTs upon the chemical transformation of encapsulated molecules are considered. In Section 9, a discussion of the modification of the electronic properties of filled SWCNTs is presented. In Section 10, applications of metallocene-filled SWCNTs in electrochemistry, thermoelectric power generation, chemical sensors, and magnetic recording are discussed.

## 2. Growth Mechanism of Carbon Nanotubes

### 2.1. Physical State of Catalyst

**Figure 2:**
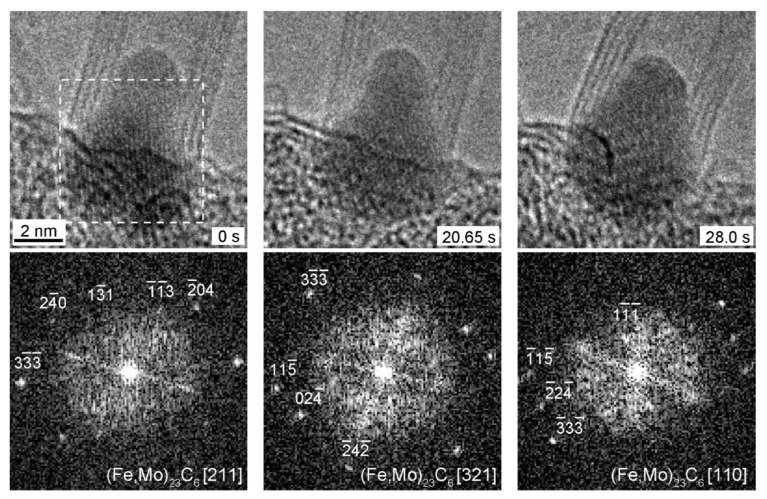
In situ environmental TEM observation of the growth of a multi-walled carbon nanotube from a fluctuating nanoparticle of a (Fe,Mo)_23_C_6_-type structure. The recording time is shown in each image. Fourier transform of the dotted square region in each image is shown in the lower row. All the lattice images and spots are consistently accounted for by the fluctuating (Fe,Mo)_23_C_6_-type structure. Reprinted with permission from Yoshida, H. et al. Atomic-Scale Analysis on the Role of Molybdenum in Iron-Catalyzed Carbon Nanotube Growth. *Nano Lett.* **2009**, *9*, 3810–3815. Copyright 2009 American Chemical Society [95].

The authors of Ref. [88] suggest that, for the majority of high-yield CVD techniques such as injection methods for growing SWCNTs at temperatures in the order of 1000 °C [89,90], the catalyst is likely to be in the liquid state. However, in situ transmission electron microscopy (TEM) observations on the growth of SWCNTs and MWCNTs on metallic and carbidic nanoparticles at temperatures up to 650 °C demonstrated that the particles remained crystalline during the growth process, although the particles changed their shape [91,92,93,94,95]. It was shown that Ni nanoparticles with a size down to ~4–5 nm stayed crystalline at temperatures as high as 540 °C [91] and 615 °C [92] while they were growing carbon nanotubes. The authors of Ref. [93] observed structural fluctuations in a solid Fe_3_C nanoparticle at 600 °C that was growing an SWCNT with a diameter as small as 1.5 nm. In another instance, they observed the growth of a ~15–20 nm diameter MWCNT on the surface of a crystalline Fe_3_C nanoparticle. The crystal structure of the active catalyst particle was found to be fluctuating between Fe_23_C_6_ and Fe_3_C structures with random crystallographic directions, indicating that carbon atoms were migrating through the bulk (Figure 2) [95].

### 2.2. Chemical State of Catalyst

The formation of metal carbides from pure metals and their subsequent decomposition prior to nanotube growth has been confirmed several times [96,97,98,99]. This finding strongly suggests the decomposition of metal carbides as a crucial step of carbon nanotube synthesis [99]. Iron carbide was observed immediately before the start of nanotube growth [97], and the onset of growth coincided with the decomposition of the carbide to Fe and graphite [96]. Time-resolved XPS studies of catalyzed nanotube synthesis showed chemisorbed carbon and carbidic carbon on the Fe catalyst during the incubation phase before nanotube growth; once the growth commenced, the peak of the sp^2^ graphitic carbon network emerged [98].

**Figure 3:**
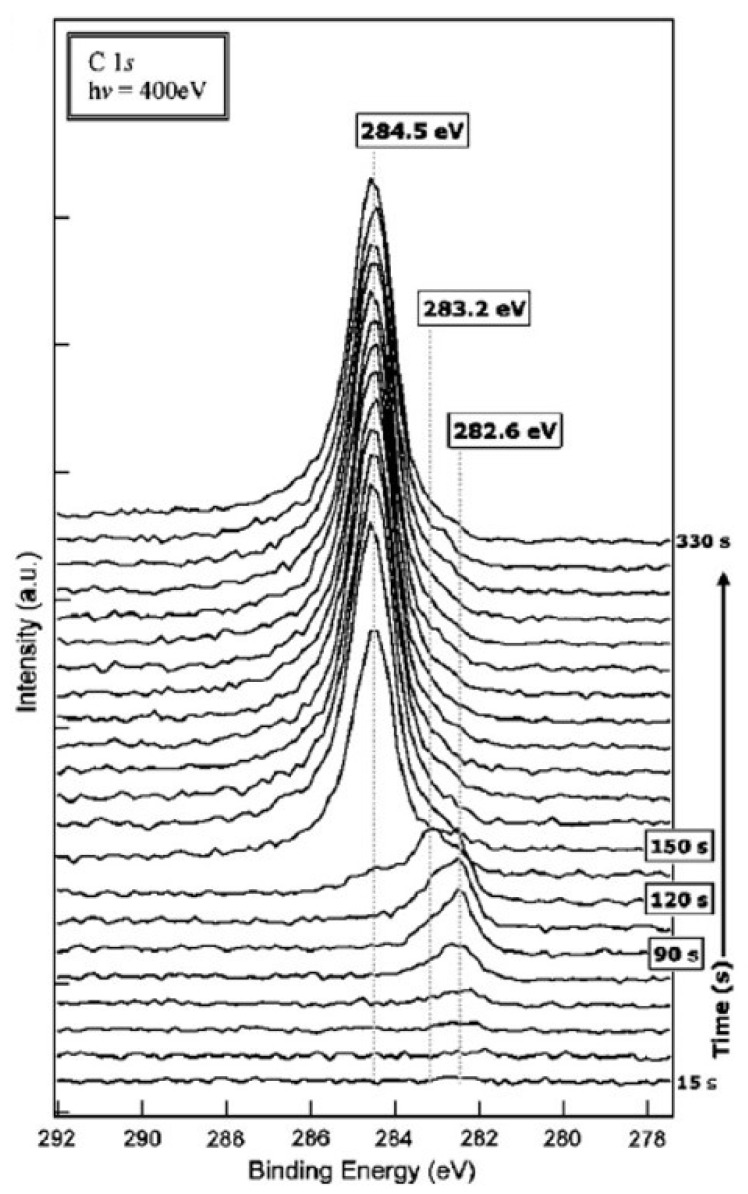
Series of C 1s in situ XPS spectra during the CVD growth of SWCNTs. The spectra (normalized to the photon flux) are recorded every 15 s. The dotted vertical lines indicate the increasing binding energies of chemisorbed, carbidic, and sp^2^ carbon. Reprinted from Mattevi, C. et al. Surface-bound chemical vapour deposition of carbon nanotubes: In situ study of catalyst activation. *Physica E* **2008**, *40*, 2238–2242, Copyright 2008, with permission from Elsevier [98].

Figure 3 shows the time-resolved evolution of the C 1s XPS peak [98]. As soon as C_2_H_2_ is let into the chamber, the peak at 282.6 eV signifies that carbon is chemisorbed on the Fe catalyst. After 90 s of incubation, a carbidic carbon peak at 283.2 eV persists for 30 s. The formation of an sp^2^ carbon network heralds the appearance of another peak at 284.5 eV. The graphitic peak quickly dominates over the other two carbon species before it saturates after 60 more seconds, when nanotube growth stops.

It should be noted that intermediate carbide phases are in many cases not directly confirmed by in situ TEM analysis of nanotube growth on a nickel catalyst [91,92,100]. The distinction of nickel and nickel carbide is very challenging because the Ni atoms have the same structure with very similar lattice constants, and thus they cannot be easily distinguished by diffraction or imaging [88]. The distinction becomes especially hard if partial carburization below the surface the catalyst particle is considered [101]. However, in situ TEM has been successfully applied to directly see the structures of iron and cobalt carbides in active catalyst particles during the growth of nanotubes [94,95,102,103,104,105].

**Figure 4:**
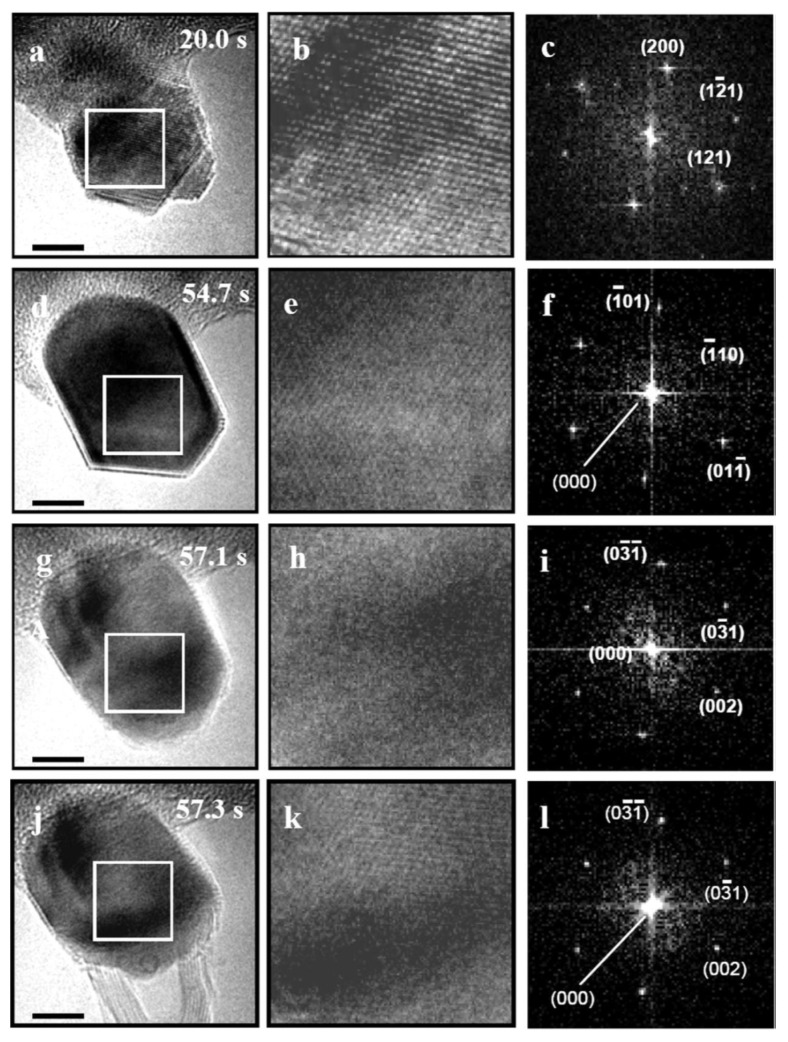
Rows of a high-resolution overview image (**a**,**d**,**g**,**j**), a closeup of the boxed region in the overview (**b**,**e**,**h**,**k**), and the diffractogram of the closeup (**c**,**f**,**i**,**l**), all extracted from the same digital video sequence. The bar was 5 nm. The deposited Fe particle was heated to 650 °C under 10 mTorr of flowing C_2_H_2_. At 20 s (**a**–**c**) the irregular Fe particle had a bcc structure. At 54.7 s (**d**–**f**) the Fe particle was a bcc crystallite with {110} faces. At 57.1 s (**g**–**i**) the rounded particle consisted of Fe_3_C. By 57.3 s (**j**,**k**,**l**) the particle had grown a multiwall carbon nanotube and still consisted of Fe_3_C. Reprinted with permission from Sharma, R. et al. Site-Specific Fabrication of Fe Particles for Carbon Nanotube Growth. *Nano Lett.* **2009**, *9*, 689–694. Copyright 2009 American Chemical Society [103].

Figure 4 shows rows of a high-resolution TEM image overview, a closeup of the boxed region in the overview, and the diffractogram of the closeup at different times from a continuous video sequence [103]. The Fe particle was heated to 650 °C and exposed to 10 m Torr of flowing C_2_H_2_. The deposited particle had an irregular shape and a bcc structure. After 34.7 s, the particle was a crystallite of bcc iron with {110} faces. After 37.1 s, the particle had a more rounded shape, the diffractogram confirming Fe_3_C. By 37.3 s, the particle had already grown a multiwall carbon nanotube and its diffractogram proves it was still made of Fe_3_C.

### 2.3. Tip- and Base-Growth Models

Base-growth was observed for MWCNTs in Refs. [94,106,107] and for SWCNTs in Refs. [92,94,100,108,109]. Tip-growth was reported for MWCNTs in Refs. [91,92,93,102] and for SWCNTs in Refs. [110,111,112]. Time-resolved in situ HRTEM is arguably the most direct tool for investigating the growth of carbon nanotubes and for distinguishing the tip- and base-growth mechanisms [91,92,93,94,100,110].

**Figure 5:**
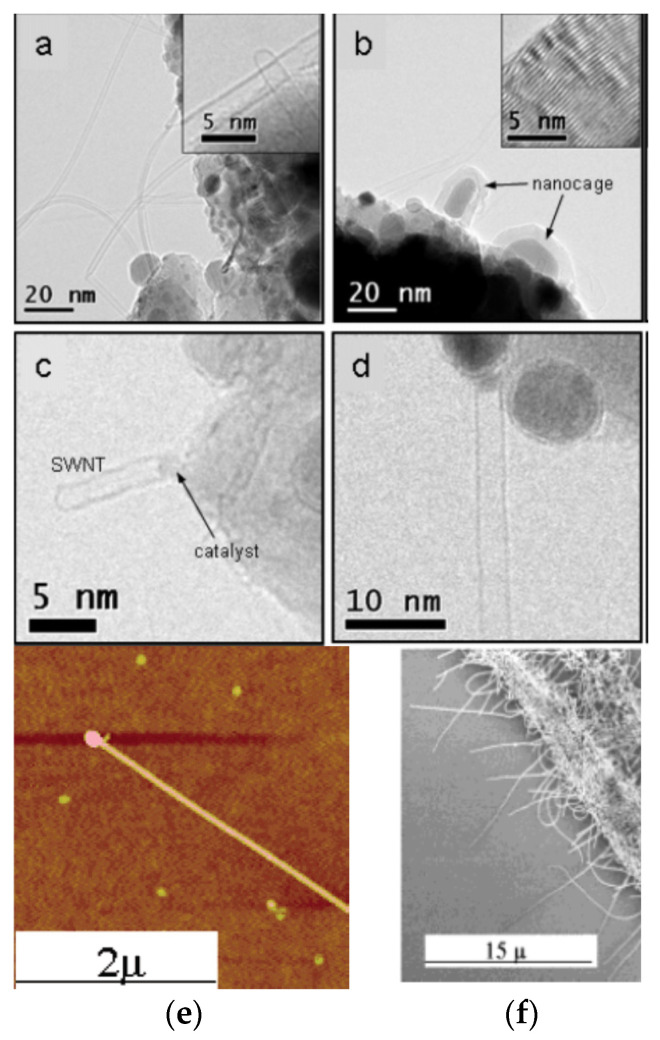
TEM images acquired after growth. (**a**) Bundled SWNTs; (**b**) nanocages; (**c**,**d**). Reprinted with permission from Lin, M. et al. Direct Observation of Single-Walled Carbon Nanotube Growth at the Atomistic Scale. *Nano Lett.* **2006**, *6*, 449–452. Copyright 2006 American Chemical Society [100]. (**e**) AFM image of a long oriented nanotube with a particle at the tip, obtained by a fast-heating CVD process. Reprinted with permission from Huang, S. M. et al. Growth Mechanism of Oriented Long Single Walled Carbon Nanotubes Using “Fast-Heating” Chemical Vapor Deposition Process. *Nano Lett.* **2004**, *4*, 1025–1028. Copyright 2004 American Chemical Society [112]. (**f**) SEM image of random short SWNTs from a conventional growth process using Fe/Mo nanoparticles as catalysts and CO/H2 as a feeding gas at 900 °C for 10 min. Reprinted with permission from Huang, S. M. et al. Growth Mechanism of Oriented Long Single Walled Carbon Nanotubes Using “Fast-Heating” Chemical Vapor Deposition Process. *Nano Lett.* **2004**, *4*, 1025–1028. Copyright 2004 American Chemical Society [112].

Figure 5a–d shows TEM images of SWCNTs and nanocages [100]. The diameters of the SCWNTs range between 0.6 and 3.5 nm. Their lengths vary between a few nanometers and one micrometer. All SWCNTs had clean straight walls and closed Ni catalyst-free tips, which is indicative of base-growth. The diameters of Ni catalyst particles at the base are correlated to the diameter of the SWCNT.

Base-growth was frequently observed under typical synthesis conditions for carbon nanotubes [92,94,100,108,109]. There are also well documented scenarios where tip-growth occurs in SWCNTs [110,111,112]. In Ref. [112], a fast-heating CVD process was employed to synthesize long and aligned SWCNTs. Careful inspection confirmed that both tip- and base-growth mechanisms had occurred during the synthesis. Moreover, the long and oriented nanotubes were produced exclusively by the tip-growth mechanism. Figure 5f shows the SEM image of the short random SWCNTs, obtained in a classical CVD process [112]. Direct evidence for the tip-growth mechanism in long and aligned SWCNTs under fast-heating conditions came from AFM imaging. The tips of all long aligned SWCNTs featured a nanoparticle. One example is shown in Figure 5e [112]. The size of the particles at the tip of the nanotubes was in most cases slightly larger than the SWCNT’s diameter. The authors of Ref. [112] argue that the catalyst particles likely grow due to amorphous carbon deposition during the cooling process.

### 2.4. Tangential and Perpendicular Growth Modes

Besides the tip- and base-growth mechanisms there is also the distinction between tangential and perpendicular growth modes [113,114,115].

TEM data was employed to conduct a statistical analysis of the correlation between the diameters of SWCNTs and the sizes of the catalytic nanoparticles on which they grow [113]. The images unanimously proved the existence of two types of nanotube nuclei for the tangential and perpendicular growth modes, respectively. In the tangential growth mode, the diameter of the nanotube is close to that of the nanoparticle. In the perpendicular growth mode, the diameter of the carbon nanotube is much smaller than that of the particle and the carbon walls have a nearly perpendicular contact angle to the surface of the nanoparticle [113]. The diameters of nanotubes nucleated in the perpendicular growth mode are not correlated with that of the nanoparticle. If the diameter of the nanotube is less than 75% of the particle diameter it can only have been formed by the perpendicular growth mode. The ratio of tangential to parallel nucleations is not affected by the diameter of the particles. It is, however, affected by the synthesis time.

## 3. Characterization of the Structure of SWCNTs Filled with Molecules

This section is dedicated to analysis of the structure of SWCNTs filled with metallocenes, and the structure obtained from them due to chemical reactions.

### 3.1. Scanning Transmission Electron Microscopy

**Figure 6:**
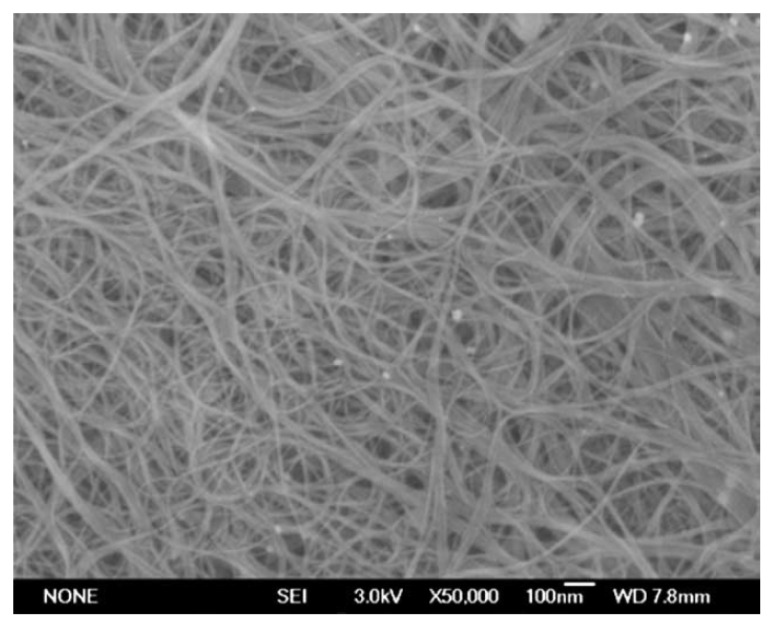
SEM image of SWCNTs filled with ferrocene. Reprinted from Guan, L. et al. Ferrocene-filled single-walled carbon nanotubes. *Carbon* **2005**, *43*, 2780–2785, Copyright 2005, with permission from Elsevier [54].

Figure 6 presents a scanning transmission electron microscopy (SEM) image of ferrocene-filled SWCNTs [54]. The image shows bundles of filled SWCNTs, which are homogeneously located throughout the sample. The image proves no impurities of excess ferrocene on the outer side of the carbon nanotubes’ walls, which confirms the successful filling of the channels of the carbon nanotubes with ferrocene.

### 3.2. Transmission Electron Microscopy

#### 3.2.1. Ferrocene-Filled Carbon Nanotubes

**Figure 7:**
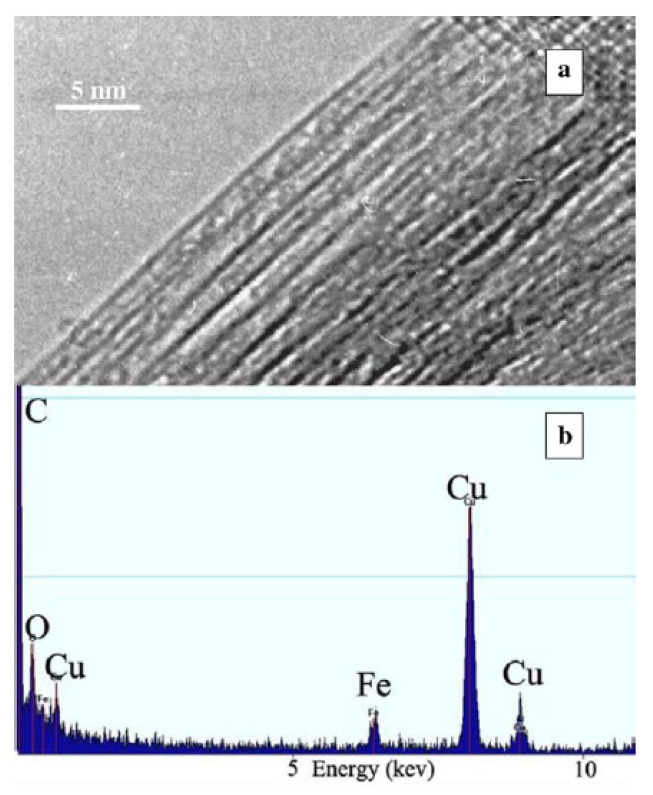
(**a**) TEM image of bundle of ferrocene-filled SWCNTs. (**b**) The EDX analysis of filled SWCNTs. Reprinted from Guan, L. et al. Ferrocene-filled single-walled carbon nanotubes. *Carbon* **2005**, *43*, 2780–2785, Copyright 2005, with permission from Elsevier [54].

Figure 7a shows a TEM image of ferrocene-filled SWCNTs [54]. The image shows the bundle of filled carbon nanotubes. It is visible that the interior space inside the SWCNTs on the edge of the bundle is filled with molecules. The energy-dispersive analysis (EDX) (Figure 7b) shows the presence of iron peaks in the spectrum, which corresponds to the filling of the carbon nanotubes [54].

**Figure 8:**
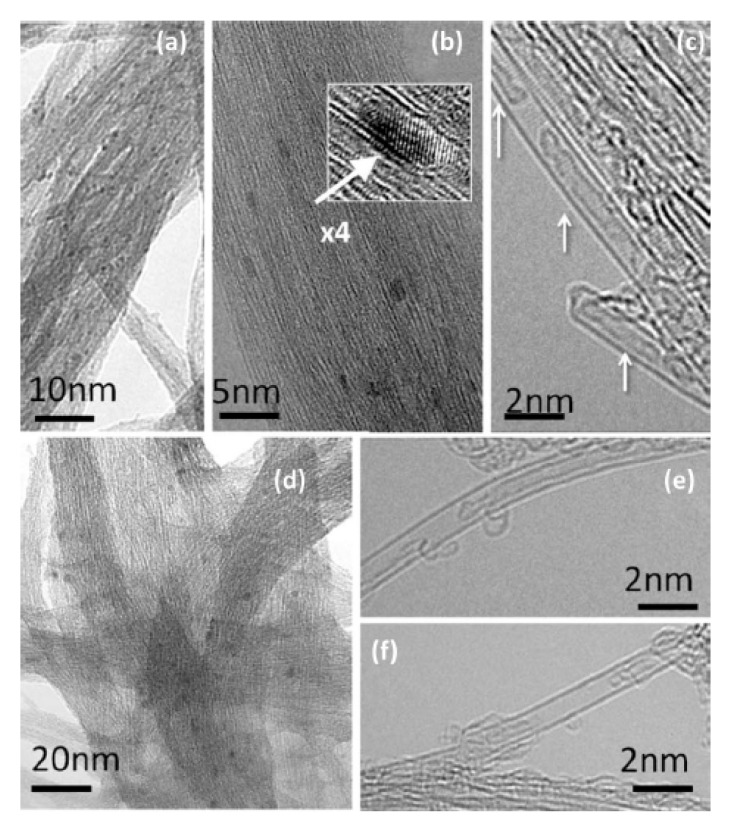
(**a**–**c**) TEM image of iron/iron carbide-filled semiconducting SWCNTs in low (**a**), intermediate (**b**), and high (**c**) magnifications. The inset in (**b**) shows the metallic cluster. (**d**–**f**) The TEM image of filled metallic SWCNTs in low (**d**), intermediate (**e**), and high (**f**) magnifications. Reprinted figure with permission from [117] as follows: Briones-Leon, A. et al. Orbital and spin magnetic moments of transforming one-dimensional iron inside metallic and semiconducting carbon nanotubes. *Phys. Rev. B* **2013**, *87*, No. 195435. Copyright 2013 by the American Physical Society [117].

In Ref. [117], the iron/iron carbide clusters were formed inside carbon nanotubes due to the annealing of ferrocene-filled carbon nanotubes. Figure 8 shows the metal cluster-filled carbon nanotubes. Figure 8a–c shows the filled semiconducting SWCNTs, with the inset in Figure 8b showing the individual cluster. Figure 8d–f shows the filled metallic SWCNTs, which are filled with iron.

**Figure 9:**
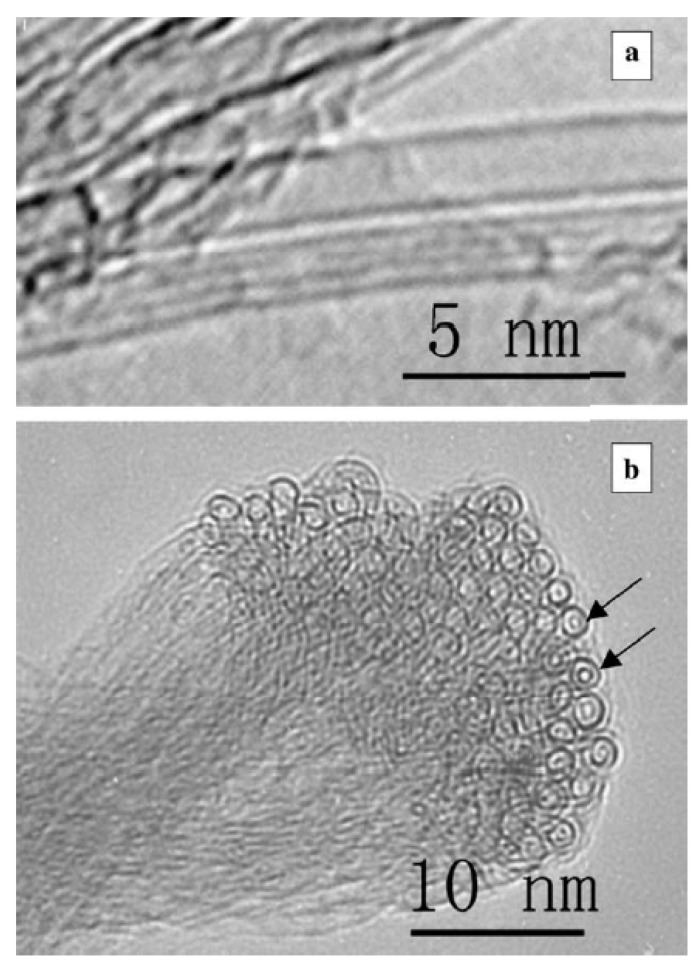
(**a**) TEM image of an individual DWCNT and SWCNTs. (**b**) Image of the cross-section of a bundle of carbon nanotubes. Reprinted from Guan, L. et al. Ferrocene-filled single-walled carbon nanotubes. *Carbon* **2005**, *43*, 2780–2785, Copyright 2005, with permission from Elsevier [54].

The thermal treatment of ferrocene-filled carbon nanotubes can lead to the formation of double-walled carbon nanotubes (DWCNTs). Figure 9 shows a TEM image of an individual DWCNT and the cross-section of a bundle of DWCNTs [54]. The presence of two walls is clearly visible in the image. Figure 9a shows the individual DWCNT and a nearby SWCNT for comparison. Figure 9b shows the cross-section of carbon nanotubes.

#### 3.2.2. Cobaltocene-Filled Carbon Nanotubes

**Figure 10:**
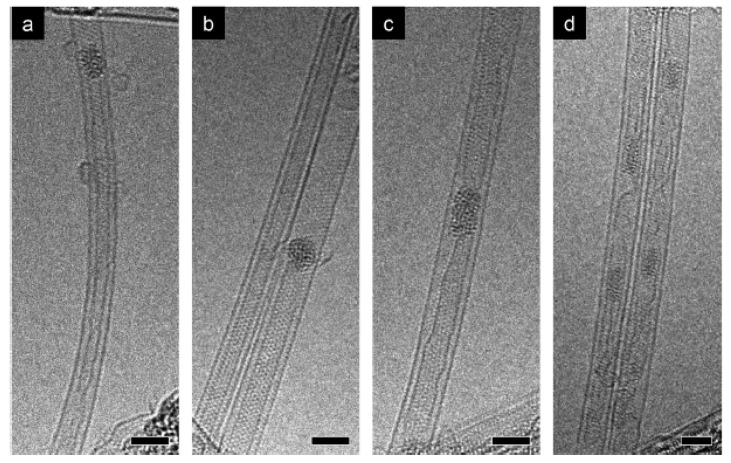
TEM images of cobaltocene-filled SWCNTs annealed at 550 °C for 2 h. (**a**) Individual DWCNTs with cluster of metal. (**b**) Two DWCNTs with metallic clusters. (**c**) A forming individual DWCNT. (**d**) Two SWCNTs that are forming DWCNTs with fillings inside them. Reprinted from Kharlamova M.V. et al. Chiral vector and metal catalyst-dependent growth kinetics of single-wall carbon nanotubes. *Carbon* **2018**. *V.133*. P.283–292, Copyright 2018, with permission from Elsevier [72].

The structure of cobaltocene-filled carbon nanotubes was studied by TEM imaging. The thermal treatment of filled SWCNTs was conducted to obtain cobalt/cobalt carbide clusters inside carbon nanotubes. Figure 10 shows the metal-filled carbon nanotubes obtained after the annealing of cobaltocene-filled SWCNTs at 550 °C for 2 h [72]. The images show the presence of metal clusters inside the carbon nanotubes.

**Figure 11:**
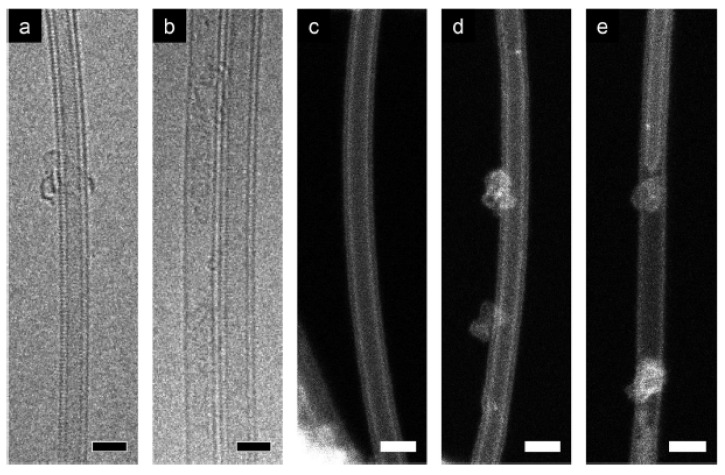
TEM and scanning TEM images of cobaltocene-filled SWCNTs annealed at 800 °C for 2 h. (**a**) Individual DWCNT. (**b**) DWCNT and forming DWCNT. (**c**) Image of DWCNT. (**d**) Individual growing DWCNT. (**e**) Growing DWCNT. Reprinted from Kharlamova M.V. et al. Chiral vector and metal catalyst-dependent growth kinetics of single-wall carbon nanotubes. *Carbon* **2018**. *V.133*. P.283–292, Copyright 2018, with permission from Elsevier [72].

Figure 11 shows TEM images of cobaltocene-filled SWCNTs annealed at 800 °C for 2 h [72]. The DWCNTs are formed in all images. Figure 11a shows the clean DWCNT. Figure 11b shows the DWCNTs and nearby SWCNTs forming DWCNTs. Figure 11c–e shows scanning TEM images of DWCNTs. Figure 11c shows a clean individual DWCNT. Figure 11d,e shows growing DWCNTs.

#### 3.2.3. Nickelocene-Filled Carbon Nanotubes

**Figure 12:**
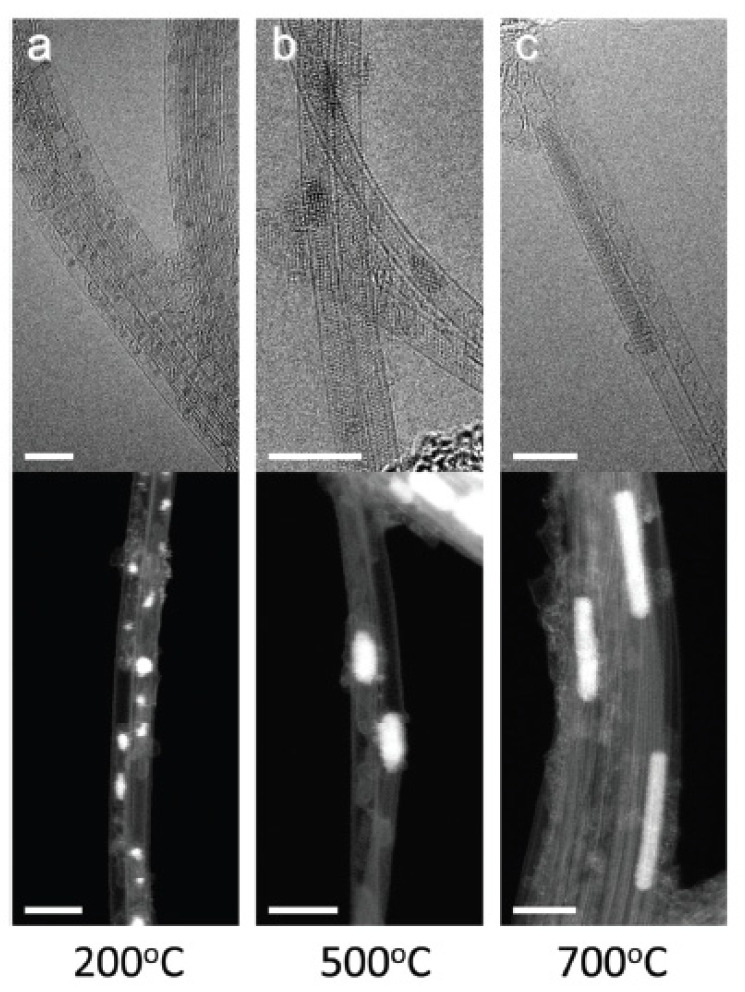
TEM and scanning TEM images of nickelocene-filled SWCNTs annealed at 200 °C (**a**), 500 °C (**b**), 700 °C (**c**) for 2 h. Reproduced from Ref. [75] with permission from the Royal Society of Chemistry. This article is licensed under a Creative Commons Attribution 3.0 Unported Licence [75].

Nickelocene molecules were filled inside SWCNTs, and TEM was used to investigate the structure of the filled SWCNTs and annealed filled carbon nanotubes. Figure 12 shows TEM and scanning TEM images of nickelocene-filled SWCNTs annealed at 200 °C (Figure 12a), 500 °C (Figure 12b), 700 °C (Figure 12c) for 2 h [75]. It is visible that the increase in annealing temperature leads to the formation of longer metallic clusters.

## 4. Growth Processes of Filled Carbon Nanotubes and Other Structures

**Figure 13:**
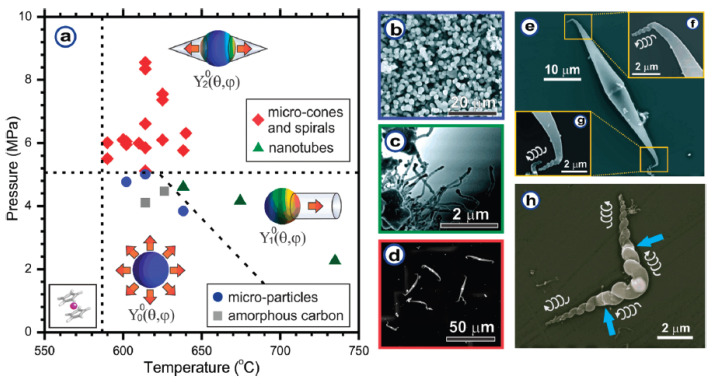
(**a**) Phase diagram for the growth process of synthesized structures depending on the pressure and temperature of synthesis. SEM images of microparticles (**b**), nanotubes (**c**), microcones, and spirals in different positions (**d**–**h**). Reprinted with permission from Shiozawa, H. et al. Spontaneous Emergence of Long-Range Shape Symmetry. *Nano Lett.* **2011**, *11*, 1, 160–163. Copyright 2011 American Chemical Society [119].

In Ref. [119], the treatment of ferrocene at different conditions (pressure, temperature) was shown to lead to the formation of amorphous carbon, microparticles, nanotubes, microcones, and spirals. The processes occurred due to self-organization. Figure 13a shows the phase diagram for the growth process of synthesized structures depending on the pressure and temperature of synthesis [119]. At pressures above 5 MPa and temperatures above 590 °C, the mirrored structure microcones and spirals were observed. At higher temperatures and lower pressures, the formation of nanotubes was observed. At low temperatures and pressures, amorphous carbon was formed. Figure 13b–h shows SEM images of amorphous carbon, microparticles, nanotubes, microcones, and spirals [119].

## 5. Characterization of the Kinetics of the Growth of Carbon Nanotubes

The growth kinetics of carbon nanotubes was characterized in Refs. [72,75,120]. It was shown that the growth process of carbon nanotubes is characterized by two activation energies, E_α_ and E_β_, of growth on carbide and metallic catalytic particles, accordingly. These two stages are characterized by two growth rates, α and β. The growth process depends on the diameter of the nanotube and the type of metal.

**Figure 14:**
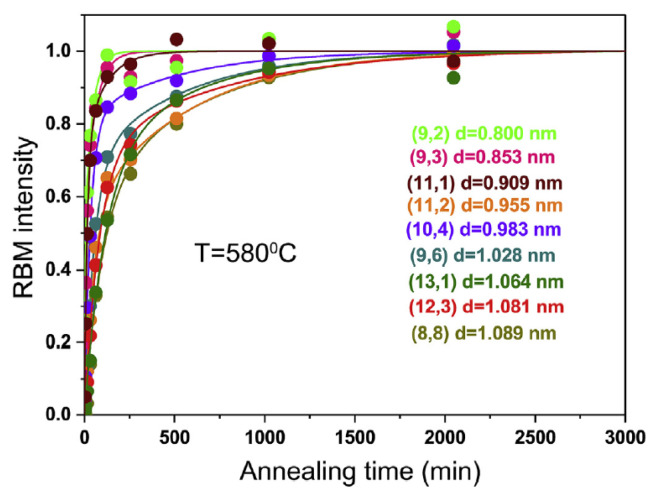
The dependence of the radial breathing mode (RBM) intensity on the annealing time for carbon nanotubes with different chiralities growing on a cobalt catalyst at 580 °C. Reprinted from Kharlamova M.V. et al. Chiral vector and metal catalyst-dependent growth kinetics of single-wall carbon nanotubes. *Carbon* **2018**. *V.133*. P.283–292, Copyright 2018, with permission from Elsevier [72].

Figure 14 shows the growth curves of carbon nanotubes with different chiralities at 580 °C on a cobalt catalyst [72]. It is visible that the carbon nanotubes grow quickly with rate α at the beginning of the growth process. They continue to grow slower with rate β. Here the annealing times from 2 min to 3000 min are considered, and saturation is achieved.

**Figure 15:**
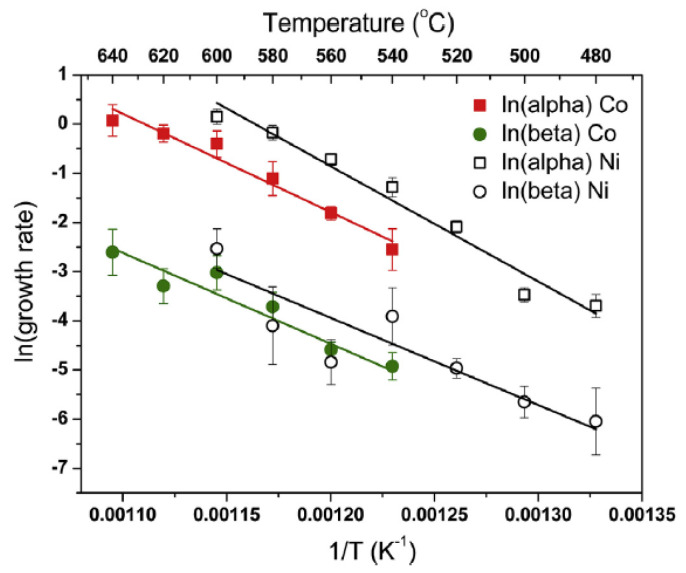
Logarithmic plots of rates α and β of the growth of carbon nanotubes with different chiralities on cobalt and nickel catalysts. Reprinted from Kharlamova M.V. et al. Chiral vector and metal catalyst-dependent growth kinetics of single-wall carbon nanotubes. *Carbon* **2018**. *V.133*. P.283–292, Copyright 2018, with permission from Elsevier [72].

Logarithmic plots of growth rates α and β were prepared using the experimental data on the growth of carbon nanotubes with different chiralities on cobalt and nickel catalysts (Figure 15) [72]. They exhibit linear behavior. The temperature range from 480 °C to 640 °C is considered, and it is shown in the upper abscissa axis. The arrows for every spot, which were considered for the fitting of the linear function, are shown.

**Figure 16:**
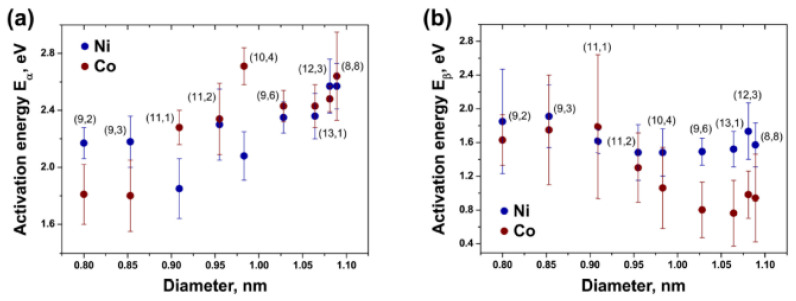
The dependence of activation energies E_α_ (**a**) and E_β_ (**b**) on the diameter of the nanotube. Copyright 2021 by the authors. Licensee MDPI, Basel, Switzerland. This article is an open access article distributed under the terms and conditions of the Creative Commons Attribution (CC BY) license [120].

Figure 16 shows the dependence of activation energies E_α_ (Figure 16a) and E_β_ (Figure 16b) on the diameter of the nanotube [120]. The authors demonstrated that activation energy E_α_ decreases with decreasing the carbon nanotube diameter for both the nickel and cobalt catalysts. Activation energy E_β_ shows only a small dependence on the diameter. The authors skipped a discussion of the dependence of the activation energy on the chiral angle.

The following nine points in the observed dependences should be refined.

The activation energy of growth in carbide and metal particles decreases when the tube diameter is decreased. In the case of the activation energy of growth in carbide particles, the activation energy is larger for cobalt than nickel for larger-diameter tubes, and it is larger for nickel than cobalt for smaller-diameter tubes. This is caused by differences in the bulk structures of nickel and cobalt particles. The value for a (10,4) carbon nanotube grown on a cobalt catalyst is the maximum because of the structure of the carbon tube. The value for a (9,3) carbon nanotube grown on a cobalt catalyst is the minimum because of the structure of the carbon nanotube. In the case of the activation energy of growth in metallic particles, the activation energy is larger for a nickel catalyst than a cobalt catalyst for larger-diameter tubes, and it is larger for a cobalt catalyst than for a nickel catalyst for smaller-diameter carbon nanotubes. The value for a (12,3) carbon nanotube is the minimum for a cobalt catalyst, and the value for several carbon nanotubes (12,3), and (9,3), are maximal for a nickel catalyst among other carbon nanotubes, because of their atomic structure.

An increase in activation energy for a cobalt catalyst is caused by the modification of its atomic structure from hexagonal to cubic and then monoclinic.

An increase in activation energy for a nickel catalyst is caused by the modification of its atomic structure from cubic to hexagonal and then to monoclinic. This required additional theoretical calculations for the optimization of the crystal structure of catalyst particles.

The chiral angle’s dependence on the activation energy of growth in carbon nanotubes for both catalysts (nickel and cobalt) exists in a torch-like shape on the plot, which is caused by the different atomic structure and gradual change in chiral angle of the carbon nanotube when it grows on catalyst particles at a different angle relative to the walls of the carbon nanotube (see figure). Here on the figure we see that nanotubes with the largest chiral angles have the largest activation energies of growth. The nanotubes with smaller chiral angles have smaller activation energies, but they are positioned in a range from maximal to minimal. The nanotubes with the smallest chiral angles have the smallest activation energies. The nanotubes (10,4), (9,3), and (12,3) have the largest activation energies due to their structures, and this leads to a broadening of chiral angle distributions. The distributions are not shown here, because they are broadened.

The chiral angle’s dependence on the activation energy is broadened, but it is visible that cobalt particles have higher activation energies for the growth of carbon nanotubes.

The growth rates of carbon nanotubes decrease when the nanotube diameter is increased, with the exception being the chiral angle’s dependence on growth, which is observed to be the same for both activation energies.

The growth rate increases for a nickel catalyst as compared to a cobalt catalyst because of differences in the diffusion rates of metal and carbon.

For a fixed-time growth, the growth rate of the carbon nanotube increases with when the tube diameter is decreased; this dependence is refined with chiral angle dependence, which is, as discussed in the above-mentioned points, due to its dependence on the activation energy of grown carbon nanotubes. It is important to note that this point should be refined.

For the dependence of fixed-time growth on temperature, the growth rate of the carbon nanotube decreases for larger-dimeter armchair carbon nanotubes, which have the largest diameter in the case of nickel and cobalt catalysts. The growth rates of carbon nanotubes increase for zigzag tubes, which can have smaller diameters.

For fixed-time growth, the growth rates are larger for a nickel than a cobalt catalyst. They have different diffusion rates for different chiral angles of chosen carbon nanotubes. This should be refined by the experiment and theoretical calculations.

Here, we discuss the points of the activation energy and growth rate dependence of carbon nanotubes for chosen data for nickelocene- and cobaltocene-filled SWCNTs. The results can vary for other data, but the discussed tendencies provide a fundamental theory with respect to the growth kinetics and growth dynamics of carbon nanotubes. This theory can be formulated in five points:

- The growth of carbon nanotubes depends on the temperature and time.
- The growth of carbon nanotubes depends on the metal catalyst and precursor.
- The growth rate depends on the temperature and metal catalyst type.
- The growth rate depends on the diffusion rate of metal and carbon.
- The activation energy depends on the tube diameter and chiral angle, because of structural differences between the catalyst and carbon nanotube.

## 6. Characterization of the Optical, Vibronic, and Electronic Properties of SWCNTs Filled with Molecules

### 6.1. Filling of SWCNTs with Molecules

Organic and organometallic (metallocene and metal acetylacetonate) molecules decompose at high temperatures. Powders of these substances cannot be melted, but they can be sublimed in vacuum at elevated temperatures without decomposing the molecules. The gas phase method was employed to fill SWCNTs for spectroscopic investigations of their modified electronic properties. In the gas phase method, the SWCNTs and an excess amount of the organic or organometallic powder are sealed in a quartz ampoule. One half of the ampoule is heated to gradually re-sublime the heated powder from the warm side onto the cooler side. The buckypaper of the SWCNTs is positioned in the center. The heated and unheated ends of the ampoules are typically flipped twice a day and the entire gas phase filling takes several days.

This method has allowed for SWCNTs to be filled with ferrocene [53,56,59,60,61,63,65,66], cobaltocene [3,71,72], nickelocene [73,74,75], cerocene [77,78], and acetylacetonates of Pt (II) [64,79,80] and Ni (II) [81]. It should be noted that the incorporation of ferrocene [62] and Pt (II) acetylacetonate [64,79] into SWCNTs has also been conducted via the liquid phase method using their solutions in acetone.

### 6.2. Investigation of Doping Effects in Metallicity-Mixed SWCNTs Filled with Molecules

In the literature, investigations of metallocene-filled SWCNTs by Raman spectroscopy, near edge X-ray absorption fine structure spectroscopy (NEXAFS), photoemission spectroscopy (PES), and optical absorption spectroscopy (OAS) have been performed [121,122,123,124,125,126,127,128,129,130,131,132,133,134,135,136,137,138,139,140,141,142,143,144,145,146,147,148,149,150,151,152,153,154,155,156,157,158,159,160,161].

#### 6.2.1. Photoemission Spectroscopy

Information on changes in the bonding environment and the Fermi level in molecule-filled SWCNTs was obtained by photoemission spectroscopy. In the literature, the C 1s XPS spectra of SWCNTs filled with cerocene [77,78], ferrocene [59,61,65,67], nickelocene [73], cobaltocene [3], and nickel acetylacetonate [81] molecules have been reported. Modifications in the electronic properties of the filled SWCNTs are reflected in the shifted position, altered width, and changed shape of the C 1s peak. The filling of SWCNTs with molecules usually leads to an upshift in the C 1s binding energy and the increased asymmetry and width of the C 1s peak. These changes testify to a lower work function due to the charge transfer from the incorporated molecules to the SWCNTs.

**Figure 17:**
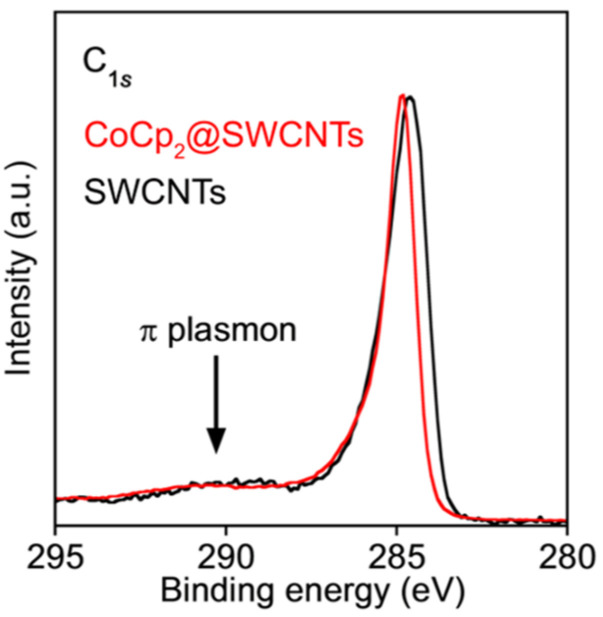
C 1s XPS spectra of pristine and cobaltocene-filled SWCNTs. The p-plasmon is indicated. Reproduced from [3]. This work is licensed under a Creative Commons Attribution-NonCommercial-NoDerivs 4.0 International License.

Figure 17 shows the C 1s XPS spectra of the pristine and cobaltocene-filled SWCNTs [3]. Comparison of the spectra reveals an upshift in the C 1s peak, its increased asymmetry and a decrease in the intensity of the shake-up peaks at ~291 eV (denoted by the arrow) due to the electronic interaction between the nanotubes and the introduced cobaltocene.

In Ref. [63], the determination of the shift value in the Fermi level of the ferrocene-filled SWCNTs was performed by UPS. In the valence band spectra of the filled SWCNTs, the peak positions of the first and the second vHs of semiconducting SWCNTs and the first vHs of metallic nanotubes were consistently upshifted by 0.05 eV, which is equivalent to increasing the Fermi level of SWCNTs by this value. This n-doping is due to the charge transfer from the encapsulated molecules to the SWCNTs.

#### 6.2.2. Optical Absorption Spectroscopy

**Figure 18:**
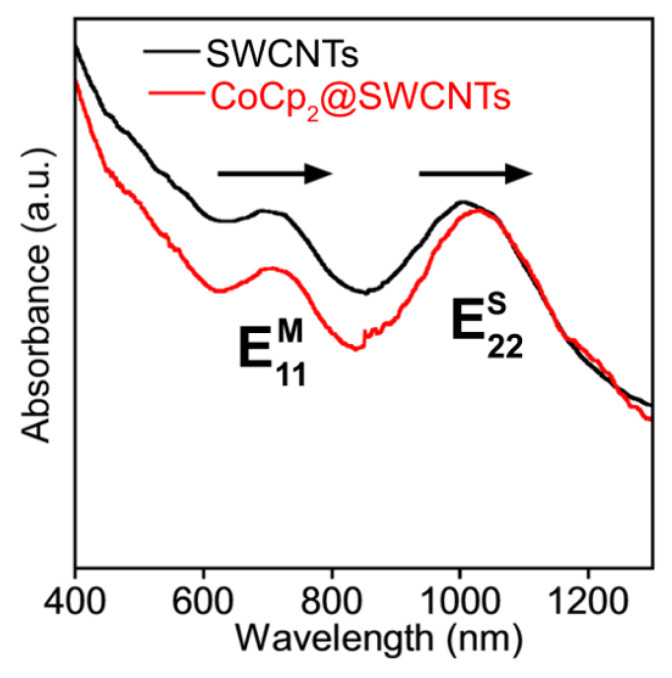
OAS spectra of the pristine and cobaltocene-filled SWCNTs. The S_22_ and M_11_ absorption bands are denoted. The red-shifts of the bands are shown by arrows. Reproduced from [3]. This work is licensed under a Creative Commons Attribution-NonCommercial-NoDerivs 4.0 International License.

The charge transfer in molecule-filled SWCNTs was studied by optical absorption spectroscopy. Figure 18 demonstrates the OAS spectra of the pristine and cobaltocene-filled SWCNTs with a mean diameter of 1.4 nm [3]. The spectra reveal the characteristic $E_{22}^{S}$ and $E_{11}^{M}$ absorption bands of semiconducting and metallic SWCNTs positioned at wavelengths around 900–1300 nm and 600–800 nm, respectively. Both absorption bands are red-shifted by ~30 meV in the case of the filled SWCNTs, which was explained by n-doping in the SWCNTs by the encapsulated cobaltocene [3].

In Ref. [57], the OAS spectra of pristine, open-ended, and ferrocene-filled SWCNTs with a mean diameter of 1.0 nm were obtained. Comparison of the spectra of the pristine SWCNTs and open-ended SWCNTs showed notable differences in the S11 region. In the latter, the peaks of the largest-diameter SWCNTs in the range from 900 to 1100 nm were suppressed and red-shifted due to p-doping and the improved screening of optical excitons [57]. After filling the SWCNTs with ferrocene, the peaks in the S11 region maintained the red shift, but their intensity increased significantly. This is due to the unchanged screening of optical excitons and the charge transfer from the incorporated ferrocene molecules to the SWCNTs, i.e., n-doping, that can even overcome the ambient p-doping in the pristine SWCNTs and increase peak intensities [57].

## 7. Investigation of Doping Effects on Metallicity-Sorted SWCNTs Filled with Molecules

In the literature, there are reports on the comparison of the doping effect of molecules on metallicity-sorted metallic and semiconducting SWCNTs. In Ref. [67], a comprehensive spectroscopic investigation of the charge transfer in the ferrocene-filled metallic and semiconducting SWCNTs was conducted by combining XAS, XPS, and UPS.

**Figure 19:**
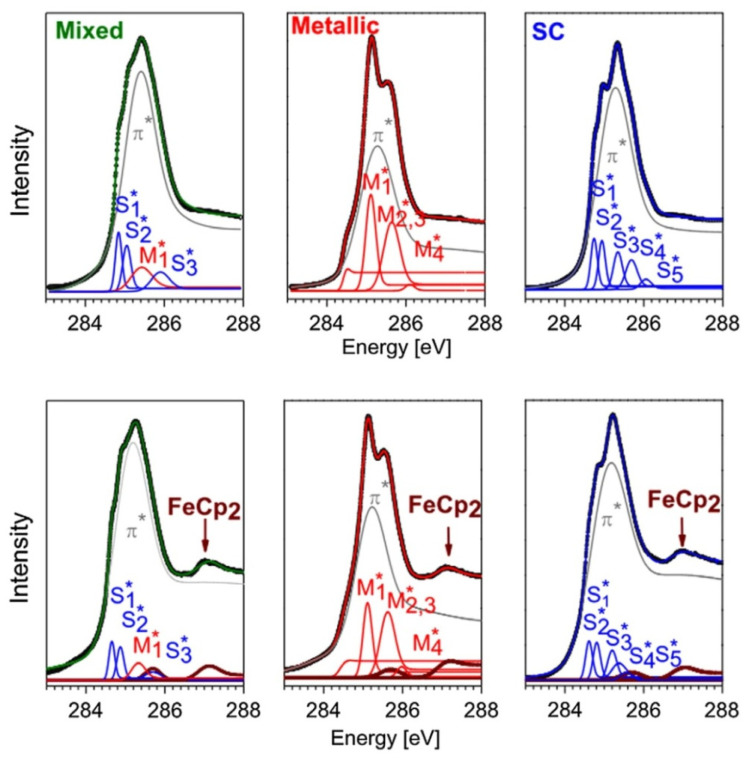
C 1s XAS spectra of 1.4 nm-diameter pristine (**top** panels) and ferrocene-filled (**bottom** panels) metallic, semiconducting (SC), and metallicity-mixed SWCNTs fitted with individual components. The experimental data are shown as black circles. The fit data for the metallicity-mixed, metallic, and semiconducting SWCNTs are shown in green, red, and blue, respectively. The data are fitted with the π*-peak (grey) and the peaks of individual vHs of metallic ($M_{1}^{*}–M_{4}^{*}$) (red) and semiconducting ($S_{1}^{*}$–$S_{5}^{*}$) SWCNTs (blue). In the filled SWCNTs, two peaks in the C 1s XAS response from pure ferrocene are indicated (dark red). Reprinted from Sauer M. et al. Internal charge transfer in metallicity sorted ferrocene filled carbon nanotube hybrids. *Carbon* **2013**. *V.59*. P.237–245, Copyright (2013), with permission from Elsevier [67].

Figure 19 shows the C 1s XAS spectra of the 1.4 nm-diameter pristine and ferrocene-filled metallic, semiconducting, and metallicity-mixed SWCNTs [67]. The spectra include the π*-resonance, originating from an electronic transition from the C 1s core level to the unoccupied π*-conduction band; it demonstrates fine features, which correspond to an electronic transition from the C 1s core level to individual vHs in the conduction band of SWCNTs. The C 1s XAS spectra are fitted with a broad π*-peak at photon energies ranging from 284 to 286 eV and several components of individual vHs. For the metallic SWCNTs, the components of vHs are positioned at energies of 0.7 eV ($M_{1}^{*}$), 1.25 eV ($M_{2,3}^{*}$) and 1.7 eV ($M_{4}^{*}$) above the C 1s absorption edge. For the semiconducting SWCNTs, the components of vHs are located at energies of 0.40 eV ($S_{1}^{*}$), 0.60 eV ($S_{2}^{*}$), 1.00 eV ($S_{3}^{*}$), 1.35 eV ($S_{4}^{*}$), and 1.70 eV ($S_{5}^{*}$) above the absorption edge. For the metallicity-mixed SWCNTs, whose electronic structure is a result of a complex interplay between the metallic and semiconducting SWCNTs, the components of vHs are shifted as compared to those of the metallicity-sorted SWCNTs. The components of vHs of the semiconducting and metallic SWCNTs are positioned at energies of 0.50 eV ($S_{1}^{*}$), 0.70 eV ($S_{2}^{*}$), 1.00 eV ($M_{1}^{*}$), and 1.55 eV ($S_{3}^{*}$) above the C 1s absorption edge.

In the case of the ferrocene-filled SWCNTs, there is a decrease in the intensity of the components of vHs of the metallic and semiconducting SWCNTs in comparison to the π*-peak, as well as a change in their relative intensities (Figure 19) [67]. In the case of the metallic SWCNTs, the component of the $M_{1}^{*}$ vHs in particular decreases in intensity as compared to the other ones, whereas the relative intensities of the components of all other vHs are kept constant. In the case of the semiconducting and metallicity-mixed SWCNTs, there is a slight decrease in the relative intensity of the components of the $S_{1}^{*}$ and $S_{2}^{*}$ vHs as compared to the component of the $S_{3}^{*}$ vHs in the semiconducting SWCNTs and the component of the $M_{1}^{*}$ vHs in the metallicity-mixed SWCNTs. In all spectra of the filled SWCNTs, two peaks in the C 1s XAS response from pure ferrocene are present. The authors of Ref. [67] explained the observed trends by the charge transfer from the encapsulated ferrocene to the nanotubes and hybridization between π-orbitals of the carbon in the SWCNTs and the molecules.

**Figure 20:**
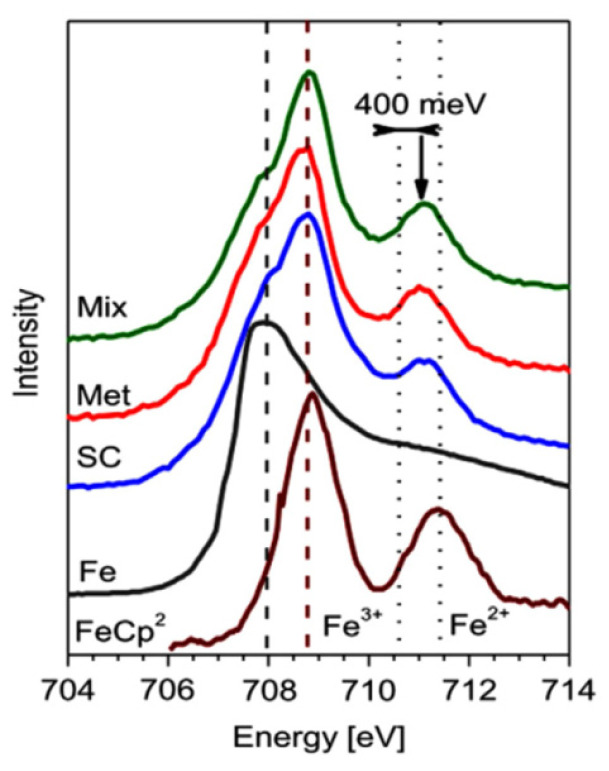
Shows the Fe2p_3/2_–Fe 3d XAS spectra of ferrocene (FeCp_2_), bulk Fe, and mixed (Mix), metallic (Met), and semiconducting (SC) ferrocene-filled SWCNTs. The line positions of bulk Fe and ferrocene are marked with dashed vertical lines. The position of the secondary peak depends on the Fe valency. The dotted vertical lines mark its positions for valency states of +3 and +2. Reprinted from Sauer M. et al. Internal charge transfer in metallicity sorted ferrocene filled carbon nanotube hybrids. *Carbon* **2013**. *V.59*. P.237–245, Copyright (2013), with permission from Elsevier [67].

The doping effects in the ferrocene-filled metallic and semiconducting SWCNTs were also investigated by XAS at the Fe edge. The XAS spectrum of the ferrocene-filled nanotubes was compared to the spectra of metallic Fe and pure ferrocene. Figure 20 shows the Fe2p_3/2_–Fe 3d XAS edges [67]. There are two different components resolved in the spectra of pure ferrocene and filled SWCNTs. The stronger absorption peak is positioned around 708.75 eV; it is blue shifted as compared to elemental Fe, where it is found at 708 eV. The strongest peak belongs to transitions to molecular orbitals (MO) of FeCp_2_ from Fe 3d_xz_ and Fe 3d_yz_ together with a small p*(Cp)-ligand contribution. The weaker peak at around 711.1 eV belongs to transitions to the p*(Cp) MOs from Fe 3d_xy_ and Fe 3$d_{x^{2}-y^{2}}$, which corresponds to a metal-to-ligand transition [67].

**Figure 21:**
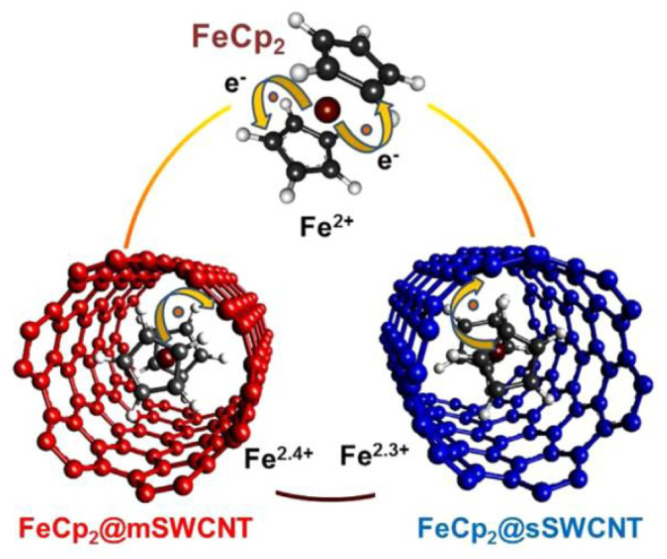
Schematic drawing of the charge transfer and associated Fe valency in ferrocene and ferrocene-filled metallic (FeCp_2_@mSWCNT) and semiconducting SWCNTs (FeCp_2_@sSWCNT). Reprinted from Sauer M. et al. Internal charge transfer in metallicity sorted ferrocene filled carbon nanotube hybrids. *Carbon* **2013**. *V.59*. P.237–245, Copyright (2013), with permission from Elsevier [67].

The red-shift of this peak to lower photon energies as compared to pure ferrocene is attributed to an increase in the electron-nuclear Coulomb attraction, which corresponds to a higher valency state [67]. The effective valency of iron in the ferrocene-filled SWCNTs is determined by the peak positions. The effective valencies of Fe in ferrocene inside metallic and semiconducting SWCNTs are +2.4 and +2.3, respectively. The different valencies are illustrated in the schematic in Figure 21 [67].

These valencies of Fe in ferrocene inside SWCNTs are much bigger than the +2 valency in pure ferrocene, where one electron is transferred onto each of the cyclopentadienyl rings. This directly quantifies the n-doping of metallic and semiconducting SWCNTs by the encapsulated ferrocene. The charge transfer is more effective for the ferrocene-filled metallic SWCNTs [67].

## 8. Investigation of Doping Effects on Metallicity-Mixed and Sorted SWCNTs upon Chemical Transformation of Encapsulated Molecules

Molecules can undergo chemical transformations inside the SWCNT channels, which leads to the modification of the electronic properties of nanotubes. The types of chemical reactions involved may include a simple thermal decomposition as well as more complex chemical transformations. The authors of Ref. [80] demonstrated a variety of chemical reactions that can be conducted in the interior of SWCNTs starting from Pt (II) acetylacetonate as precursor. SWCNTs are filled with Pt (II) acetylacetonate via the gas phase approach at 150 °C in high vacuum. Then, Pt (II) acetylacetonate can be decomposed at 500 °C with the formation of pure Pt clusters inside SWCNTs. The former react with iodine, leading to the formation of platinum iodide inside the SWCNTs. Pt (II) acetylacetonate can be inserted in the interior of nanotubes simultaneously with iodine at 150 °C in high vacuum. The thermal treatment of the mixture formed inside the SWCNT channels at 500 °C leads to the formation of platinum iodide. Finally, platinum iodide can react with sulfur at 550 °C to form platinum sulfide. The SWCNTs can also be filled with trans-bis(acetylacetonato)di-iodoplatinum Pt(acac)_2_I_2_ and bis(acetylacetonato)di-thiocyanatoplatinum Pt(acac)_2_(SCN)_2_ via the gas phase method at 130–150 °C in high vacuum. The thermal treatment of the compounds inside the SWCNTs leads to the formation of platinum iodide and platinum sulfide, respectively. The authors of Ref. [80] studied the direction of electron transfer in the filled SWCNTs by tracing the position of the G-band peak in the Raman spectra. They concluded that there is no electron transfer in SWCNTs filled with Pt (II) acetylacetonate, platinum iodide, and platinum sulfide. In the case of SWCNTs filled with pure Pt and Pt(acac)_2_(SCN)_2_, electron transfer from the compounds to the SWCNTs, i.e., n-doping, was revealed. In the case of SWCNTs filled with Pt(acac)_2_I_2_, electron transfer from the SWCNTs to the compound, i.e., p-doping, was observed.

The most popular group of chemical reactions conducted inside molecule-filled SWCNTs that lead to the modification of their electronic properties is the thermal decomposition with the formation of inner tubes. Annealing is a feasible way to control chemical transformations in the incorporated molecules and to control the effect of the encapsulated substance on the electronic properties of nanotubes. After the precursor molecules have decomposed, there is a local supply of carbon that can be gradually molded into inner nanotubes, which again actively participate in charge transfer. By choosing the precursor molecules and the temperature and duration of its processing, the nanochemical reaction can be fine-tuned for a stable ambipolar doping level in SWCNTs.

It has been demonstrated that the thermal treatment of different molecules inside SWCNTs can lead to the formation of inner tubes: fullerenes (C_60_ [32,33,162,163,164,165,166,167,168,169] and C_70_ [32,33]), endohedral fullerenes (Gd@C_82_ [37,40,42]), metallocenes (ferrocene [53,55,56,57,58,60,61,62,63,64,65,68], cerocene [77,78], nickelocene [73,74,75,76], cobaltocene [71,72]), metal acetylacetonates (Pt(acac)_2_ [64,79], Ni(acac)_2_ [81]), and other molecules (toluene+ C_60_ [170], anthracene [171]).

**Figure 22:**
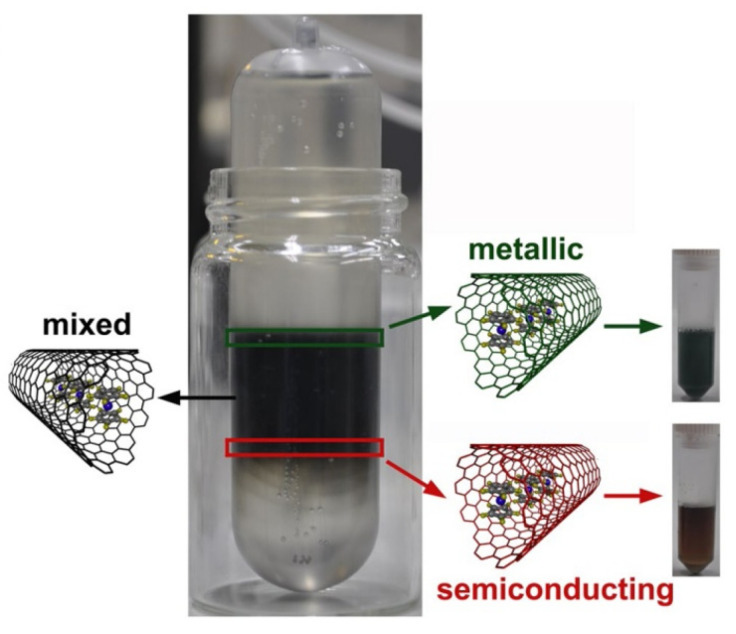
Schematic of the sorting of nickelocene-filled SWCNTs to metallic and semiconducting fractions. Reproduced from Kharlamova M.V. et al. Inner tube growth and electronic properties of metallicity-sorted nickelocene-filled semiconducting single-walled carbon nanotubes. *Appl. Phys. A*. *V.124*. N.3. article number 247, 2018, Springer Nature [76].

In Ref. [76], the thermal treatment of the nickelocene-filled semiconducting SWCNTs with a mean diameter of 1.7 nm at temperatures between 360 and 1200 °C caused a change in the doping level of nanotubes and even a switching of the doping type. The nickelocene-filled semiconducting SWCNTs were obtained by the density gradient ultracentrifugation of the filled SWCNTs, and, as a result of the centrifugation process, a semiconducting fraction was obtained in the bottom part of the centrifugation tube (Figure 22) [76]. The thermal treatment of the nickelocene-filled semiconducting SWCNTs led to the chemical transformation of the nickelocene into nickel carbides and metallic nickel at low annealing temperatures (360–600 °C), the formation of inner tubes at temperatures higher than 600 °C, and the simultaneous evaporation of nickel from the nanotubes, which caused in unison the variation in the doping level of SWCNTs [76].

**Figure 23:**
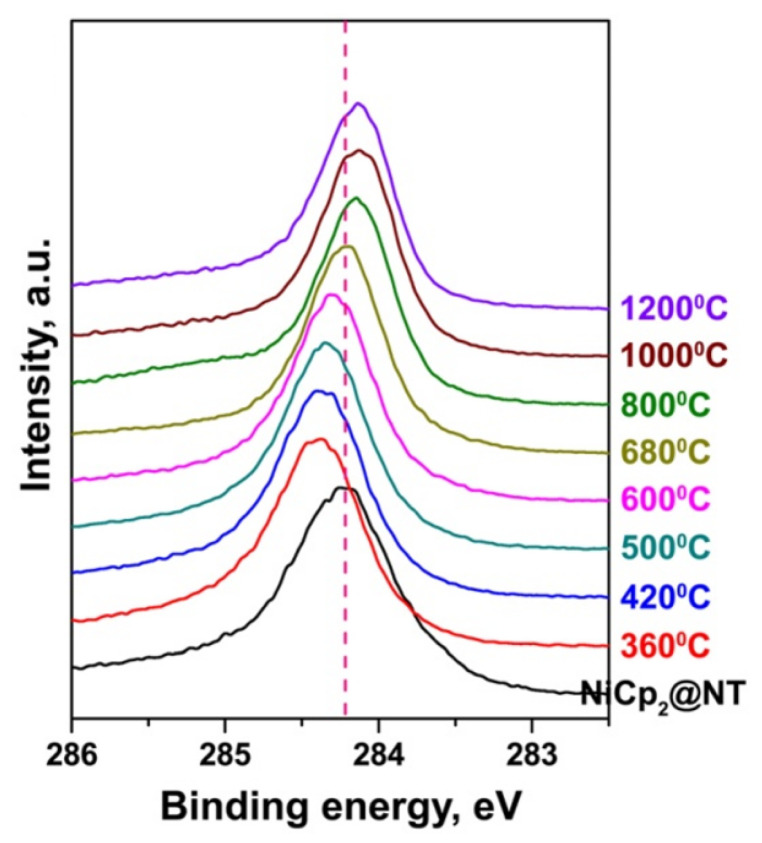
C 1s XPS spectra of nickelocene-filled semiconducting SWCNTs and the samples annealed at temperatures between 360 and 1200 °C. Reproduced from Kharlamova M.V. et al. Inner tube growth and electronic properties of metallicity-sorted nickelocene-filled semiconducting single-walled carbon nanotubes. *Appl. Phys. A*. *V.124*. N.3. article number 247, 2018, Springer Nature [76].

Figure 23 shows the C 1s XPS spectra of the nickelocene-filled semiconducting SWCNTs and the samples annealed at temperatures between 360 and 1200 °C [76]. The spectrum of the pristine SWCNTs includes a single C 1s peak. In the case of the nickelocene-filled SWCNTs, the C 1s peak is shifted toward higher binding energies, which corresponds to the upshift in the Fermi level of SWCNTs, i.e., n-doping. The shift in the C 1s peak increases at the annealing of the filled SWCNTs at 360 °C. At an increase in annealing temperature, the shift in the C 1s peak gradually decreases, but it stays positive until 600 °C. This means that the nickel carbides and metallic nickel formed as a result of the chemical transformation of nickelocene cause n-doping in SWCNTs. At a further increase in annealing temperature, the C 1s peak shifts toward lower binding energies as compared to the nickelocene-filled nanotubes. This means that the inner tubes cause p-doping in the SWCNTs. The evaporation of nickel leaves empty DWCNTs, for which the p-doping level is maximal [76].

Analogous changes in the doping level and doping type were revealed for metallicity-mixed SWCNTs with mean diameters of 1.4 and 1.7 nm filled with nickelocene [73], ferrocene [61], and nickel (II) acetylacetonate [81] molecules.

Another demonstration of controlled ambipolar doping in SWCNTs was presented in Refs. [40,42]. Gd@C82-filled nanotubes were subjected to confined nanochemical reactions. The encapsulated endohedral fullerenes caused p-doping in the SWCNTs. The thermal treatment of the metallofullerene-filled SWCNTs led to DWCNTs filled with Gd nanowires, which was proved to cause strong n-doping in the nanotubes [40,42].

The annealing of cerocene-filled SWCNTs leads to an increase in n-doping level [77,78]. Cerocene filling in itself undergoes charge transfer, and the hosting SWCNTs are n-doped. The authors of Ref. [77] found that the thermal decomposition of cerocene inside the SWCNT channels and the subsequent growth of inner nanotubes led to an increased density of states at the Fermi level. The transition into a metallic state in cerium-containing semiconducting nanotubes left its signature in an increased screening of the photoexcited final state.

## 9. Discussion of the Modification of the Electronic Properties of Filled SWCNTs

The encapsulation of molecules inside metallicity-sorted and metallicity-mixed SWCNTs with diameters of 1.4 and 1.7 nm resulted in large filling ratios. The characterization of the filled SWCNTs by OAS, XPS, and UPS showed that the incorporated organometallic molecules (NiCp_2_, CoCp_2_, FeCp_2_, CeCp_3_, Ni(acac)_2_) cause n-doping in SWCNTs accompanied by charge transfer from the molecules to the nanotubes and an upshift in the Fermi level of SWCNTs by ~0.1 eV.

**Figure 24:**
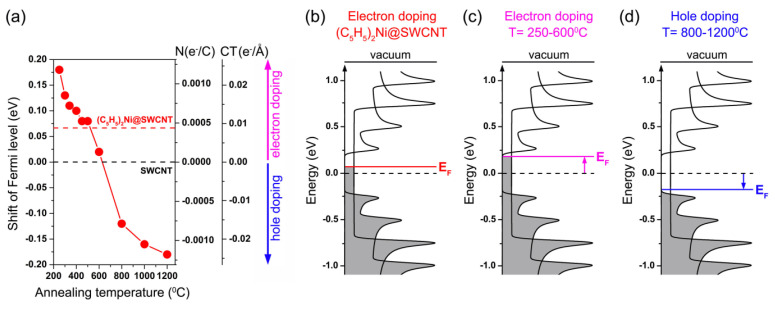
(**a**) The Fermi level shift, number of electrons transferred to carbon atom *N_total_* (e^−^ per carbon), and charge transfer density per nanotube length CT (e^−^ Å^−1^) of SWCNTs filled with NiCp_2_ upon vacuum annealing at temperatures between 250 and 1200 °C. The values of the pristine and NiCp_2_-filled SWCNTs are denoted by dashed horizontal lines. The schematics of n-doping in the NiCp_2_-filled SWCNTs (**b**) and the samples annealed at temperatures of 250–600 °C (**c**) and p-doping in the samples annealed at temperatures of 800–1200 °C (**d**). The shift in the Fermi level of the SWCNTs is shown by the arrow. Reproduced from [73]. This article is licensed under a Creative Commons Attribution 3.0 Unported Licence.

The thermal treatment of SWCNTs filled with the organometallic molecules (NiCp_2_, CoCp2, FeCp2, CeCp3, Ni(acac)2) leads to variation in the doping level of nanotubes and even a switching of the doping type from n to p due to three overlapping processes: (i) the chemical transformation of molecules with the formation of metal carbides or pure metals, (ii) the formation of inner tubes, and (iii) the evaporation of metals from the nanotubes. Figure 24 shows the shift in the Fermi level of SWCNTs filled with nickelocene upon their vacuum annealing at temperatures between 250 and 1200 °C. In Figure 24a, the doping levels for different annealing temperatures are presented. In Figure 24b–d, the schematic band structures of the NiCp2-filled SWCNTs and annealed samples are demonstrated. The annealing of the NiCp2-filled SWCNTs, where an upshift in the Fermi level as compared to the pristine SWCNTs by 0.07 eV is observed (Figure 24b), at temperatures between 250 and 600 °C leads to n-doping in the SWCNTs accompanied by an increase in the Fermi level of SWCNTs by ~0.05–0.2 eV (Figure 24c). This corresponds to the number of transferred electrons Ntotal (e^−^ per carbon) ranging from 0.00013 to 0.00118 e^−^/C and the charge transfer density per tube length CT (e^−^ Å^−1^) ranging from 0.0027 to 0.0240 e^−^/Å. The annealing of the filled SWCNTs at temperatures between 800 and 1200 °C results in p-doping in the SWCNTs accompanied by a lowering of the Fermi level of the SWCNTs by ~0.15–0.2 eV (Figure 24d), which corresponds to Ntotal ranging from −0.00118 to −0.00078 to e^−^/C and CT from −0.0240 to −0.0160 to e^−^/Å.

## 10. Applications of Filled SWCNTs

### 10.1. Electrochemistry

The authors of Ref. [172] investigated the electrochemical properties of redox active guest-molecules (cobaltocene and methylated ferrocene derivatives) inside 1.4 nm-diameter SWCNTs. It was shown that the filling inside the SWCNTs modifies the oxidation state of the metallocenes. The authors developed the technique for quantifying the electronic doping of SWCNTs using electrochemistry.

Cyclic voltammetry measurements were performed [172]. The Fermi level of the SWCNTs shifted in metallocene-filled carbon nanotubes. As a result, when the electric potential was applied, higher or lower energy levels were depleted in the metallocene@SWCNT hybrids. The first case is related to the n-dopant metallocene molecule. The second case is attributed to the p-dopant molecule.

Combined with density functional theory calculations, coulometry provides an accurate indication of n-/p-doping in SWCNTs [172]. The filling of redox active molecules inside carbon nanotubes leads to hybrids with complex, interesting electrochemical properties, which are different from the properties of individual carbon nanotubes and metallocenes.

Knowledge about the correlation between electron transfer, the diameter of nanotubes, and the metallicity type of carbon nanotubes leads to a better understanding of host–guest interactions. This opens new roads to tailoring the oxidation state of metallocenes and mofidications in the electronic properties of carbon nanotubes in complex intriguing hybrid nanostructures [172].

### 10.2. Thermoelectric Power Generation

The combination of mechanical strength, low thermal conductivity, and high electrical conductivity renders filled SWCNTs a very promising material for efficient flexible light-weight thermoelectric devices.

**Figure 25:**
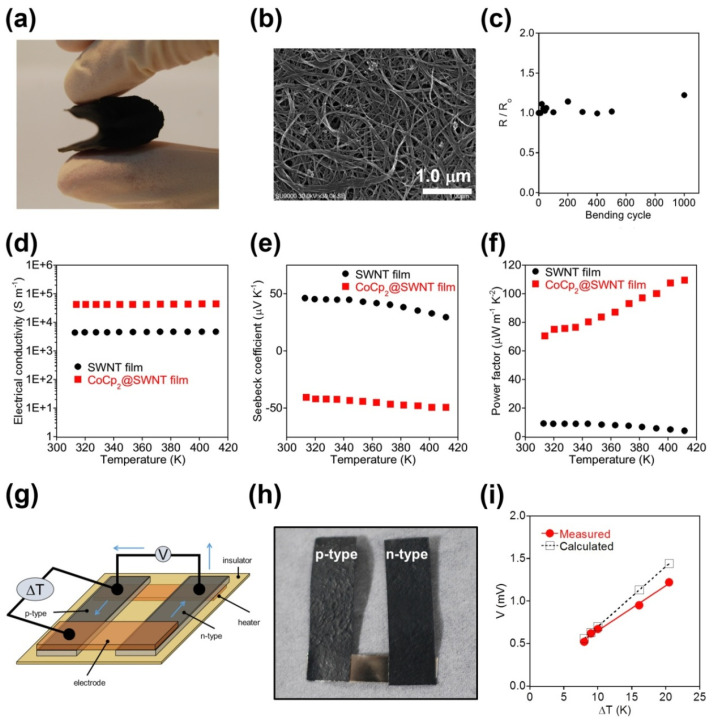
(**a**) The image and (**b**) SEM micrograph of the CoCp_2_@SWCNT film. (**c**) Normalized sheet resistance of CoCp_2_@SWCNT films plotted after repeated bending (the bending radius is 3.5 mm). R_0_ is initial resistivity, R is resistivity after given bending cycles. The electrical conductivity (**d**), Seebeck coefficient (**e**) and power factor (**f**) of the empty (black circles) and CoCp_2_-filled SWCNT films (red squares) at different temperatures. (**g**) The schematic of the setup for measuring thermoelectric power generation that consisted of the films of the CoCp_2_@SWCNT and empty SWCNTs as the *n*-type and *p*-type semiconducting materials. (**h**) The image of the thermoelectric device. (**i**) Measured (red circle) and calculated (black square) voltages (V) generated from the thermoelectric device as a function of the temperature gradient (ΔT). Reproduced from [3]. This work is licensed under a Creative Commons Attribution-NonCommercial-NoDerivs 4.0 International License.

The authors of Ref. [3] realized a flexible p-n type thermoelectric device that consisted of films of naturally p-doped empty SWCNTs and n-doped CoCp_2_-filled SWCNTs (CoCp_2_@SWCNT). The highly efficient power generation achieved approached the theoretical calculated limit and the device performed flawlessly without any air-protective coating. Two different freestanding films were fabricated and investigated. The first film consisted of CoCp_2_@SWCNT (Figure 25a) and the second film was made from empty SWCNTs. The scanning electron microscopy (SEM) micrograph in Figure 25b shows that the typical diameters of the bundles of CoCp_2_@SWCNT are in the range between 10 and 200 nm and their lengths exceed 5 μm. As shown in Figure 25c, there was no significant change in the normalized sheet resistance [3].

The electrical conductivity and Seebeck coefficients of the two types of films were measured at different temperatures (Figure 25d,e). Filling with CoCp_2_ led to an increase in electrical conductivity by one order of magnitude (from 4450 S/m for the empty SWCNTs to 43200 S/m for the filled SWCNTs at 320 K). The electrical conductivity in the films of the empty and filled SWCNTs was also found to be constant in the temperature range between 310 K and 420 K (Figure 25d).

The Seebeck coefficient of the CoCp_2_@SWCNT film amounted to a negative value of −41.8 μV K^−1^ at 320 K, which was assigned to an n-type semiconductor. The empty SWCNTs showed a positive Seebeck coefficient of 45.3 μV K^−1^ at the same temperature, which corresponds to *p*-type nature (Figure 25e). These additional electrons were provided by charge transfer from the encapsulated molecules to the host SWCNTs [3]. The higher electrical conductivity led to a significantly increased power factor of the CoCp_2_@SWCNT film (75.4 μW m^−1^ K^−2^ at 320 K) as compared to the value of the empty SWCNT film (Figure 25f).

In Ref. [3], the films of the CoCp_2_@SWCNT and empty SWCNTs as the n-type and p-type semiconducting materials were used to fabricate a p-shaped thermoelectric device. The two films were electrically connected by a thin Ni plate (Figure 25g,h). At a temperature difference of 10 K, the value amounted to 0.67 mV. This demonstrated device performance was very close to the expected value (0.70 mV) based on the Seebeck coefficients of the two films (SWCNTs: ~30 μV K^−1^, CoCp_2_@SWCNT: ~−40 μV K^−1^) [3]. The deviation from the calculated values observed at large temperature gradients was attributed to thermally-excited electrons and contact resistance (Figure 25i). However, good reversibility by decreasing the temperature gradient was observed, which was tested by applying a temperature difference of 9 K after operating the device at ΔT of 20 K [3].

### 10.3. Sensors

The working principle of SWCNT-based gas sensors is based on reversible changes in the electric properties of bundles or isolated SWCNTs when they are exposed to gases. The authors of Ref. [173] demonstrated the application potential of SWCNTs in a sensor for NO2, which is a well-known highly toxic air pollutant, capable of recovering at ambient temperature. Metallicity-sorted semiconducting and metallic SWCNTs filled with nickel (II) acetylacetonate molecules as well as nickel clusters obtained by heating at 500 °C were tested. In the case of Ni cluster-filled SWCNT, the system even fully recovered at room temperature [173].

**Figure 26:**
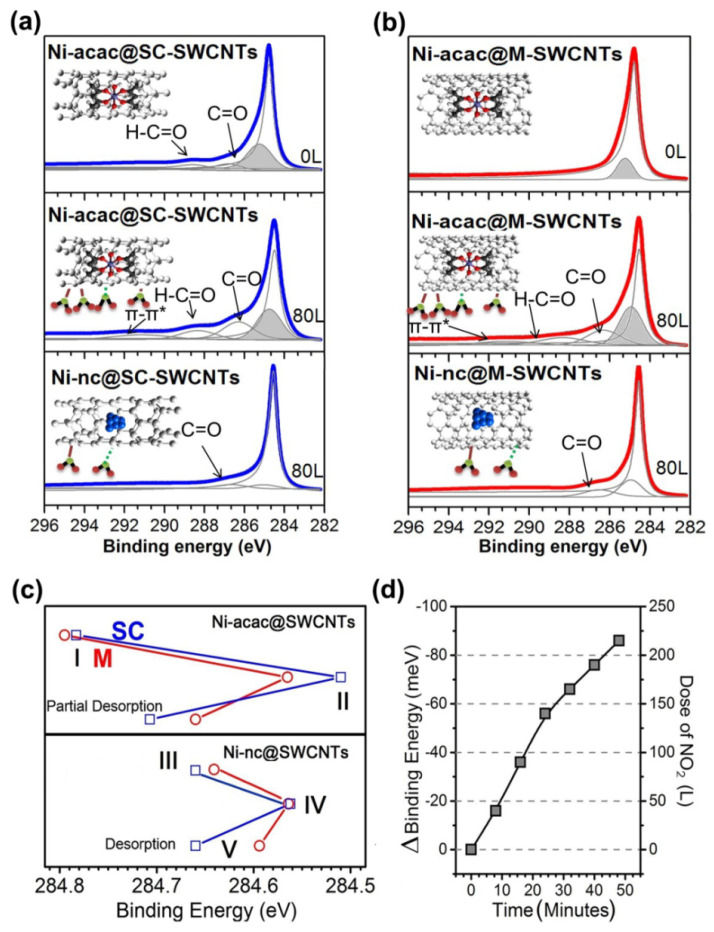
C 1s core level photoemission spectra of semiconducting (**a**) and metallic SWCNTs (**b**) filled with nickel (II) acetylacetonate molecules (Ni-acac@SC-SWCNTs and Ni-acac@M-SWCNTs, respectively) and nickel clusters (Ni-nc@SC-SWCNTs and Ni-nc@M-SWCNTs, respectively) before and after exposure to 80 L of NO_2_. The spectra are fitted with individual components of sp^2^ carbon, nickel (II) acetylacetonate filling (grey shaded), ketene group (C=O), carboxylate group (O-C=O), and π–π* interactions, which are indicated by arrows. Molecular models are presented as the inset of each plot. (**c**) The shift of the main C 1s peak of the semiconducting (SC, blue) versus metallic (M, red) hosts with different fillings at the stages I to V. (**d**) The C 1s binding energy shift in the Ni-filled semiconducting SWCNTs with increasing dose of NO_2_ over time at room temperature. Reproduced from [173]. This article is licensed under a Creative Commons Attribution-NonCommercial 3.0 Unported Licence.

Figure 26a,b shows the C 1s core level photoemission spectra from the semiconducting and metallic SWCNTs filled with nickel (II) acetylacetonate molecules (Ni-acac@SC-SWCNTs and Ni-acac@M-SWCNTs, respectively) and nickel clusters (Ni-nc@SC-SWCNTs and Ni-nc@M-SWCNTs, respectively) before and after exposure to 80 L of NO2 (1 L ≈ 1.33 · 10^−6^ mbar · s) [173]. The spectra are fitted with individual components. The dominant component around 284.5 eV in all spectra corresponds to the main carbon peak. In the case of the nickel (II) acetylacetonate filling, there is a grey shaded broad peak at slightly higher energy values as compared to the main C 1s peak, which arises from the carbon atoms associated with the nickel (II) acetylacetonate molecules. In the spectrum of nickel cluster-filled SWCNTs, this component has lower binding energy and smaller intensity, which can be associated with nickel atoms bonded to carbon atoms [173]. There are two more components at higher binding energies corresponding to two types of carbon–oxygen bonds—ketene groups (C=O) and carboxylate groups (O-C=O). The carboxylate peak is directly attributed to the nickel (II) acetylacetonate filling. The ketene groups are due to the partial oxidation of the SWCNTs during the sample preparation [173]. These components have large intensity in the spectra of the molecule-filled SWCNTs after exposure to NO2. It is also visible that these components are more pronounced for the semiconducting hosts after NO2 exposure. This suggests that the interior molecule filling increases the interaction with the external surrounding NO2. In the spectra of nickel cluster-filled SWCNTs, the carboxylate group is not observable anymore for either semiconducting or metallic hosts. It should be noted that the intensity variations and shifts are more pronounced in the semiconducting hosts, which indicates their higher sensitivity [173].

The authors of Ref. [173] studied selectivity and sensitivity by exposing the semiconducting and metallic SWCNTs filled with nickel (II) acetylacetonate molecules and nickel clusters to NO_2_ at different temperatures. The effects of the gas dosing level, the filler states, and the temperature on the C 1s core level PES spectra were investigated in situ. The in situ series for a sample were divided into five subsequent stages, I–V. In Figure 26c, the position of the main C 1s peak is traced for the filled semiconducting and metallic SWCNTs in these stages [173]. In stage I, the initial binding energies for the filled semiconducting and metallic SWCNTs cooled down to 100 K were ~284.77 and ~284.79 eV, respectively. In stage II, after exposure to 80 L of NO_2_, the C 1s binding energies downshifted by 0.26 eV and 0.23 eV for the filled semiconducting and metallic SWCNTs, respectively. This was explained by the fact that the adsorbed NO_2_ molecules acted as electron acceptors and caused a charge transfer. The samples were then left to recover without inducing changes externally. The slow desorption of the NO_2_ molecules led to the shift in the C 1s backwards (Figure 26c, top panel). It was accelerated by heating at an intermediate temperature of 400 °C to complete the desorption procedure and to obtain an adsorbent-free material [173].

Subsequently, the samples were in situ annealed at 500 °C to transform the nickel (II) acetylacetonate molecules into nickel nanoclusters inside the SWCNTs. The nickel cluster-filled SWCNTs were studied at 100 K without exposure to NO_2_. The corresponding measurements are pictured as the starting point (stage III) in the bottom panel of Figure 26c [173]. The C 1s core level PES peaks of the Ni-filled semiconducting and metallic SWCNTs have slightly different positions, which testifies to their slightly different reactivity. In stage IV, after exposure to 80 L of NO_2_, the C 1s binding energies both downshifted and coincided. This means that, although the Ni-filled semiconducting and metallic SWCNTs did not necessarily go through the same reaction pathway, they had a similar reactivity when exposed to NO_2_ [173]. When comparing the effects of NO_2_ exposure to nickel (II) acetylacetonate-filled (the stage II) and Ni cluster-filled semiconducting SWCNTs (the stage IV), one can immediately see that the molecule-filled SWCNTs were more reactive toward NO_2_, or that a physisorption process must have occurred. In such a case, the shift is assigned to charge transfer between the SWCNTs and the filler [173]. Upon the recovery of the system to reach ambient temperature at stage V in relation to III, the C 1s line position is fully restored for the Ni-filled semiconducting SWCNTs as compared to the counterpart with the metallic host tubes (Figure 26c, bottom panel). Although both Ni cluster-filled SWCNT samples reached a better recovery than the nickel (II) acetylacetonate molecule-filled SWCNTs, the authors of Ref. [173] concluded that Ni-filled metallic SWCNTs are more prone to chemisorption during exposure, while NO_2_ is mainly physisorbed on the Ni-filled semiconducting SWCNTs.

Full recovery at room temperature is a key challenge for sensors. In Ref. [173], time-resolved photoemission studies on the Ni-filled semiconducting SWCNTs were performed in order to trace the C 1s peak position upon exposure to NO_2_ at room temperature. The experiments were carried out increasing the dose of NO_2_ over 50 min. As it is shown in Figure 26d, the C 1s spectra of the Ni-filled semiconducting SWCNTs underwent a constant binding energy shift with increasing NO_2_ dosage [173]. The observed trend is consistent with the effects revealed in the above-discussed stages I to V (Figure 26c). These in situ PES studies of filled SWCNTs, which revealed remarkable results of sensitivity and recovery at ambient temperature, provide motivation for testing SWCNTs filled with appropriate substances in sensors for other reactive and poisonous gases with controlled and increased sensitivity and selectivity at room temperature.

### 10.4. Magnetic Recording

The encapsulation of substances inside SWCNTs allows for nanomagnets that outperform their bulky counterparts and control the magnetic properties of these nanohybrids to be obtained. The SWCNTs contribute their own properties to the nanohybrid, which facilitates applications of these nanomagnets in magnetorecording devices [174,175,176,177,178,179].

**Figure 27:**
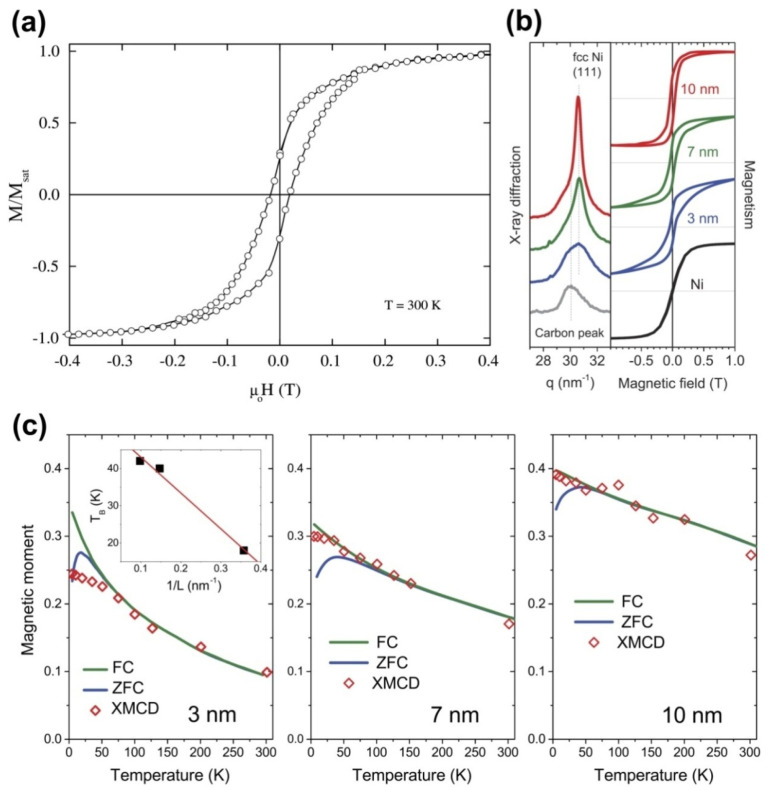
(**a**) The hysteresis loop (i.e., the magnetization (M) divided by the saturation magnetization (M_sat_) plotted versus the magnetic field (H) multiplied by the permeability of vacuum (μ_0_) for the iron-filled SWCNTs at 300 K. Reprinted from Borowiak-Palen E. et al. Iron filled single-wall carbon nanotubes—A novel ferromagnetic medium. *Chem. Phys. Lett.* **2006**. *V.421*. N.1–3. P.129–133, Copyright (2013), with permission from Elsevier [174]. (**b**) Left: X-ray diffraction profiles for the pristine SWCNTs (grey) and SWCNTs filled with 3 nm (blue), 7 nm (green) and 10 nm (red) nickel clusters, *q* is the scattering vector. The position of carbon peak and (111) fcc nickel peak are denoted by dashed vertical lines. Right: The magnetization curves for the 3 nm, 7 nm and 10 nm nickel clusters in SWCNTs and bulk nickel measured at 5 K by SQUID. (**c**) The temperature dependence of the magnetization measured upon zero–field (ZFC, blue curves) and field cooling (FC, green curves) by SQUID and normalized to the XMCD data at high temperatures. Superposed onto the SQUID data are the Ni 3*d* magnetic moments derived from XMCD data plotted versus temperature (open rectangles). The inset shows the blocking temperature (*T_B_*) plotted versus the reciprocal of the length (*L*) of the nickel cluster. Reproduced from [176]. This work is licensed under a Creative Commons Attribution 4.0 International License.

The authors of Ref. [174] encapsulated iron nanowires inside SWCNTs and revealed ferromagnetic behavior in the filled SWCNTs even at room temperature. Figure 27a shows a hysteresis loop for the Fe-filled SWCNTs at room temperature [174]. The hysteresis loop clearly evidences that the encapsulated nanowires are ferromagnetic at 300 K with a coercivity of 18 mT. The carbon shell provides effective protection from ambient oxidation [174]. In Ref. [175], behavior was ferromagnetic, whereas there was superparamagnetism at low temperatures.

Nickel nanowires with face centered cubic (fcc) structure and different sizes were formed by the thermal treatment of Ni (II) acetylacetonate in the interior of SWCNTs [176]. Its net magnetization was minimized by cooling in absence of an external magnetic field. The finite coercivity and superparamagnetic blocking temperature were found to scale with the nickel cluster size. Figure 27b shows the normalized bulk magnetization isotherms measured at 5 K by a superconducting quantum interference device (SQUID) (Figure 27b, right) for nickel clusters encapsulated in SWCNTs with mean cluster sizes of ~3 nm, 7 nm, and 10 nm evaluated from X-ray diffraction (XRD) measurements (Figure 27b, left) [176]. Upon cooling in zero field, spins of small magnetic domains are frozen to form a spin glass state with a very small net magnetization. Upon cooling in a finite field, spins are aligned, resulting in a larger net magnetization [176]. The blocking temperature TB for 3 nm, 7 nm, and 10 nm clusters equals 18, 40, and 42 K, respectively. It is inversely scaled by the cluster length (L) as illustrated in the inset in Figure 27c [176]. While SQUID is a macroscopic bulk method, XMCD is more sensitive to the nickel atoms on the surface of the clusters, because of the mean free path of low energy electrons. This applies even for the clusters with a size of 1–2 nm. The fluctuation is more visible in smaller nickel clusters. This is a manifestation of reduced dimensionality (Figure 27c) [176]. Encapsulated single-domain metal clusters have a protective carbon shell and are not affected by environmental factors. As a result, the nanohybrid can function as a stable hard magnet [176].

In Ref. [177], the magnetic susceptibility of ErCl_3_-filled SWCNTs was measured by SQUID. The magnetization of the ErCl_3_-filled SWCNTs was found to be larger than that of the purified pristine SWCNTs. The magnetization behavior of the ErCl_3_-filled SWCNTs can be fitted by the Curie–Weiss law. This value is expected from the 4f^11^ electronic configuration of Er^3+^ ions [177]. The observed magnetization behavior of the ErCl_3_-filled SWCNTs is very close to that of bulk anhydrous ErCl_3_. This means that the magnetization of Er^3+^ ions is independent of their coordination environment [177].

## 11. Conclusions, Perspectives

This review was dedicated to the electronic properties of metallocene-filled SWCNTs. It was confirmed that the ionization energy or electron affinity of molecules or the work function of elementary substances and inorganic compounds are the most important predictors of the filler’s effect on the electronic properties. They decide the sign and doping type as well as the magnitude of Fermi level shifts. The doping effect also depends on the diameter and metallicity type of the hosting SWCNT. Additionally, the filling ratio (filled vs. empty sections) directly influences the electronic properties of bulk filled SWCNT.

The growth of carbon nanotubes depends on the temperature and time, as well as on the metal catalyst and precursor. The growth rate depends on the temperature and the metal catalyst type, as well as on the diffusion rate of metal and carbon. The activation energy depends on the tube diameter and chiral angle because of structural differences between the catalyst and the carbon nanotube.

The structure of metallocene-filled SWCNTs continues to attract our interest. The development of methods of microscopy allows for the precise determination of the structure of molecules and the refinement of the molecular structure of all known metallocenes. The thermal treatment of metallocene-filled SWCNTs was first shown to lead to the formation of DWCNTs in the work of Japanese scientist Hidetsugu Shiozawa [60].

Metallocenes are a promising precursor for the synthesis of metal-filled multi-walled carbon nanotubes and SWCNTs. We demonstrated brilliant works on the synthesis of metal-filled carbon nanotubes, and investigations of the growth processes of advanced nanostructures. Such works are needed to form a fundamental insight into the chemistry of carbon nanostructures with simple chemical methods.

Investigations of the growth kinetics of carbon nanotubes develop in the direction of environmental TEM studies, in order to prove the kinetics with visual TEM images at all stages of the growth of carbon nanotubes. The experiments in the microscopes are very promising because, as shown in Section 2, they allow for the growth kinetics to be studied with a resolution of less than seconds.

The development of methods of analysis of the properties of filled carbon nanotubes allows for studying in detail the samples of metallicity sorted, single-chirality SWCNTs (which are need for applications), the precise applications of properties in devices, and the construction of devices on the basis of metallocene-filled SWCNTs. The doping effects in metallicity sorted and single-chirality SWCNTs should be studied.

Among the applications of metallocene-filled SWCNTs, those applications in electrochemistry are the most promising and recent. The thermal treatment of metallocenes leads to the formation of metal clusters. The measurement of spectra of metal clusters inside SWCNTs under electrochemical charging allows for the creation of devices with precise properties based on carbon nanotubes. The applications of metallocene-filled SWCNTs in electrochemistry open the roads to the creation of devices of metallocene-filled SWCNTs with known electrochemical properties combined with thermoelectric power generation devices, sensors, and magnetic recording devices. These devices are interesting now to investigate in projects and works. We hope that our work on the electrochemistry of carbon material is estimated positively and warmly. We do the best for the development of this topic toward the best prizes in the world [180]. We hope that referees of the project within the European Union have a warm attitude to us.

## Data Availability

Data are available on request to first author (Marianna V. Kharlamova) of every reviewed paper.
